# Verified hypotheses on the “nurse” and “burial” effects on introduced *Quercus rubra* regeneration in a mesic Scots pine forest

**DOI:** 10.1002/ece3.11185

**Published:** 2024-04-01

**Authors:** Beata Woziwoda, Marcin K. Dyderski, Anastazja Gręda, Lee E. Frelich

**Affiliations:** ^1^ Department of Geobotany and Plant Ecology, Faculty of Biology and Environmental Protection University of Lodz Łódź Poland; ^2^ Institute of Dendrology Polish Academy of Sciences Kórnik Poland; ^3^ Natural Hazards and Landscape (BFW) Austrian Federal Research Centre for Forests Vienna Austria; ^4^ Department of Forest Resources University of Minnesota St. Paul Minnesota USA

**Keywords:** acorn germination, alien tree spread, early seedling growth, northern red oak, Poland, seedling survival

## Abstract

A previous study on the encroachment of North American northern red oak *Quercus rubra* L. into the mesic Scots pine forest (in central Poland) revealed high abundances of seedlings and saplings under shrubs, with lower abundances in open areas or clumps of bilberry *Vaccinium myrtillus* L. It was unclear whether the regeneration success of *Q. rubra* is enhanced by the presence of shrubs due to their “nurse effect”, and how burying acorns of different sizes in soil or moss affects the survival of oak seeds and seedlings (a “burial effect”). Results of a previous observational study were verified in an experimental study: a pool of 900 large‐, medium‐, and small‐sized acorns was sown under moss cover in open areas and within bilberry clumps and in soil under shrubs in 2018 and monitored for 3 years in natural conditions. The majority of sown acorns were lost, mainly due to acorn pilferage, lack of germination and the death of sprouting acorns. However, acorn and seedling survival depended significantly on acorn size and differed among the microsites studied. Viable seedlings were twice as likely to develop from large‐ and medium‐sized as from small‐sized acorns, and they grew mainly from acorns sown under moss cover, confirming a positive “burial effect.” Seedling survival was three times higher in bilberry and open areas, than under shrubs; however, seedlings “nursed” by shrubs were less threatened by large ungulates. Only a small part of the pool of sown acorns contributes to the reproductive success of *Q. rubra* in the mesic Scots pine forest. Microsites characteristic to this type of forest are suitable for northern red oak regeneration; however, bilberry favors acorn survival and germination and early seedling growth, moss cover favors acorn survival and germination, while shrubs protect surviving seedlings from herbivory.

## Graphical abstract

Microsites diversity in the (common) European mesic Scots pine forests favors regeneration of introduced *Quercus rubra*. The seed “burial” under moss cover favors acorn survival and germination, while “nurse” from shrubs favors seedling survival; however, burial and nurse effects are context dependent and modified by the herbivory.

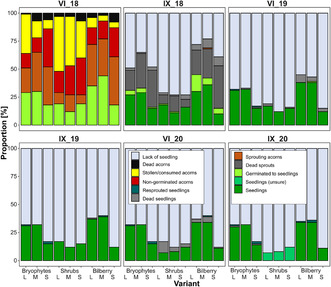

## 1 INTRODUCTION

Successful seed germination and seedling development are crucial for species survival, as the highest mortality of plants is observed in these two stages of plant regeneration (Jia et al., 2020; Mattana et al., 2023; Yan et al., 2015). In the case of commercially important woody species intentionally planted outside their native range, the successful development of juvenile specimens from seeds, naturally dispersed or collected and sown directly to the ground, also has a fundamental meaning for forest management due to the interest in less costly regeneration of stands (Löf et al., 2019, 2021; Pötzelsberger et al., 2020). Ongoing climate changes (IPCC, 2022) and predicted range shifts of native species (Koch et al., 2022; Puchałka, Paź‐Dyderska, Woziwoda, & Dyderski, 2023) additionally force the development of more effective practices for forest regeneration, also with use of introduced tree species (Badano & Sánchez‐Montes de Oca, 2022; Brang et al., 2014; Dyderski et al., 2018; Puchałka, Paź‐Dyderska, Jagodziński, et al., 2023). The abundant uncontrolled encroachment of juvenile specimens into forest patches located outside areas of alien tree cultivation, however, can be undesirable if introduced species negatively impact native ecosystems (Brundu & Richardson, 2016; Dyderski & Jagodziński, 2021; Richardson et al., 2000). The recognition of favorable environmental conditions for alien seed germination and seedling growth in different types of forest sites is essential both for the effective regeneration of introduced species as well as for control of their “too spontaneous” spread (Brundu et al., 2020).

The North American northern red oak *Quercus rubra* L. is of special interest to European foresters and ecologists. It is very common both in commercial and preserved forests, where it occurs in a wide range of forest sites (Dyderski et al., 2020; Nicolescu et al., 2018; Woziwoda et al., 2014, 2018a). Stands of *Q. rubra* produce an abundant crop of viable acorns (Gręda et al., 2022; and references therein), variable in their size and mass (Woziwoda et al., 2023), and thus attractive for different native acorn consumers which are also involved in the dispersal of oak seeds (Myczko et al., 2014; Wróbel et al., 2022). All factors mentioned above favor *Q. rubra* regeneration and its spontaneous spread (Bieberich et al., 2016; Dyderski et al., 2020). The rate and scale of colonization of new forest patches by *Q. rubra*, however, vary both for types of forest site and different microsites within the same site (Chmura, 2020; Jagodziński et al., 2018; Major et al., 2013; Miltner & Kupka, 2016; Nosko et al., 2021; Woziwoda, Dyderski, Kobus, et al., 2019). A previous study on *Q. rubra* encroachment into the mesic Scots pine (*Pinus sylvestris* L.) forest in central Poland (Woziwoda et al., 2018b) revealed abundant occurrence of northern red oak seedlings and saplings concentrated in nearby components of the forest understory (under shrubs), while fewer specimens were noted in patches without a shrub layer or within spatially extensive clumps of bilberry (*Vaccinium myrtillus* L., a dominant component of the herb layer in Scots pine forests). It was concluded that differences in the number of *Q. rubra* seedlings and saplings noted under shrubs, in the open areas and bilberry clumps, resulted mainly from a different number of acorns deposited by acorn‐hoarding animals (mainly blue jays *Garrulus glandarius* L. and rodents; Dyderski et al., 2020; Vander Wall, 1990, 2001; and references therein) in a specific type of microsite in subsequent years. The final distribution of the northern red oak juveniles, however, had to be related to different acorn and seedling “life‐stories,” conditioned, for example, by the size of a deposited acorn, post‐dispersal acorn predation, pressure from herbivores, inter‐ and/or intraspecific competition, or by other factors. The lack of information on the initial number of acorns dispersed in the forest by acorn hoarders in specific sites was indicated as the cause of possibly erroneous conclusions on the efficiency of seed germination and seedling growth in different microhabitats (Woziwoda et al., 2018b).

In the case of large‐seeded species such as *Quercus* spp., numerous studies show that the larger the acorns, the higher the percent of germinated seeds, seedling growth rate and survival rate (e.g., Bonfil, 1998; Clark et al., 2000; Ivanković et al., 2011; Kormanik, Sung, Kass, & Schlarbaum, 1998; Kormanik, Sung, Kormanik, et al., 1998; Löf et al., 2019; Tecklin & McCreary, 1991). Large acorn size usually means more reserves in cotyledons necessary for seedling growth at the early stage of its development (Long & Jones, 1996; Seiwa & Kikuzawa, 1991), so the relationships mentioned above seem obvious. However, Long and Jones (1996) and Clark and Schlarbaum (2018) revealed that neither acorn size nor mass could be used reliably as morphological indicators of seedling quality or to predict the survival of oak seedlings. Larger seeds deliver more resources at the early stages of seedling development but they do not necessarily buffer young plants from the negative effects of environmental variation (e.g., from limitations in nutrients or water) or competition of other plants (Jevon et al., 2021). However, larger acorns are attractive to seed consumers, so they can be more threatened by acorn predators (Buckley et al., 2006; Crawley, 2000; Merceron et al., 2017; Mezquida et al., 2021; Myczko et al., 2017). On the other hand, large‐sized seeds are often only partially damaged, so they still preserve the ability to germinate and produce viable seedlings (Branco et al., 2002; Hopper et al., 1985; Steele et al., 1993; Yi & Yang, 2010). Dispersed acorns can be pilfered (stolen) and re‐cached or consumed in large numbers by post‐dispersal acorn predators (Wang et al., 2014). To reduce pilferage, seed hoarders choose landscape fragments with specific features, for example, more open forest patches, or in contrast—forest fragments with a dense understory—, and hide the food in the moss cover, litter, or soil (Kollmann & Schill, 1996; Muñoz & Bonal, 2011; Sunyer et al., 2015). Acorn “hiding” in the soil is also used by commercial forestry during oak regeneration by direct seeding (García et al., 2002; Löf et al., 2019) or oak seedling production in forest tree nurseries (Crow, 1988; Dey & Parker, 1996). Buried seeds gain improved radicle penetration into the soil and protection from negative impacts of the weather, which favors seed germination and seedling recruitment (Bogdziewicz et al., 2020; Briggs et al., 2009; Zwolak & Crone, 2012) and is defined as a “burial effect” (García et al., 2002; García & Houle, 2005). The early growth of plants can be also favored by seed burial under a shrub canopy (Cuesta et al., 2010; Gavinet et al., 2016; Jensen et al., 2011; Pulido and Díaz, 2005), which is defined as a “nurse effect” (Castro et al., 2004; Gómez‐Aparicio et al., 2004, 2008).

We were interested in two questions; first, how does burying acorns (different in size) in the moss or soil in different types of microhabitats favor the spontaneous regeneration of northern red oak in the Scots pine forest? Second, what is the proportion of the pool of *Q. rubra* acorns sown in natural conditions that successfully germinate and develop into vital seedlings?

In this experimental study, we aimed to (i) identify the impact of the microsite on the survival and development of *Q. rubra* acorns and seedlings, (ii) compare the development of seedlings from acorns of different sizes sown in different microhabitats, and (iii) indicate the impact of external factors (weather and herbivory) on acorn germination and seedling growth and survival in the mesic Scots pine forest. We also wanted to verify two hypotheses formerly accepted as favorable for *Q. rubra* establishment and recruitment in the mesic Scots pine forest, that is, the “nurse effect” of shrubs and the acorn “burial effect.”

## 2 MATERIALS AND METHODS

### 2.1 Study area

To verify the results of previous research (Woziwoda et al., 2018b), we set up an experimental study in the same locality, in the Małyń‐Jerwonice forest complex (central Poland; 52°46′49″ N, 19°02′42″ E). The experimental plots were situated in the same forest fragment of the mesic Scots pine forest (with an area of 2.92 ha), and the sowing material (acorns) was collected from the adjacent Scots pine–northern red oak stand (for more information, see Woziwoda et al., 2018b).

The tree stand was commercial (artificially planted 70 years ago; FDB, 2018); Scots pines were distributed regularly and the light conditions under the canopy were relatively uniform. The shrub layer was sparse, composed of scattered clumps of alder buckthorn *Frangula alnus* Mill., silver birch *Betula pendula* L., and single specimens of northern red and pedunculate oaks *Quercus robur* L. The herb layer was dominated by clumps of bilberry *V. myrtillus*, and the forest floor was covered by mosses, mainly by *Pleurozium schreberi* (Willd. ex Brid.) Mitt.

The soil of the site was mineral, with sandy loams lying on top of sand subsoil, and classified as a “brunic podzol.” Soil was well drained, but mesic, that is, containing a moderate amount of moisture from precipitation. It was nutrient poor and acid (pH = 4–5) (FDB, 2018).

During the period of the experiment (from October 2017 to September 2020), the weather conditions varied (Figure S1). The extremely cold and dry late winter and early spring (February/March) in 2018, cold and dry springs (March–April) in 2019 and 2020, and extremely dry and hot summer (June–July) of 2019 were the characteristic features of the studied period (Meteo Data, 2023).

### 2.2 Experimental material and treatment design

Acorns were collected from the ground in October 2017. Only acorns that appeared to be viable, with no visible external damage (e.g., weevil exit holes or black fungi mycelium), were collected. To examine the impact of acorn size on *Q. rubra* germination and seedling growth, acorns were visually divided into three size classes: large, medium, and small (according to Kormanik, Sung, Kormanik, et al., 1998). Next, a sample of 300 acorns was randomly selected from each size class (900 in total) and sown the same day as harvested to limit changes in seed quality (e.g., in their moisture).

**FIGURE 1:**
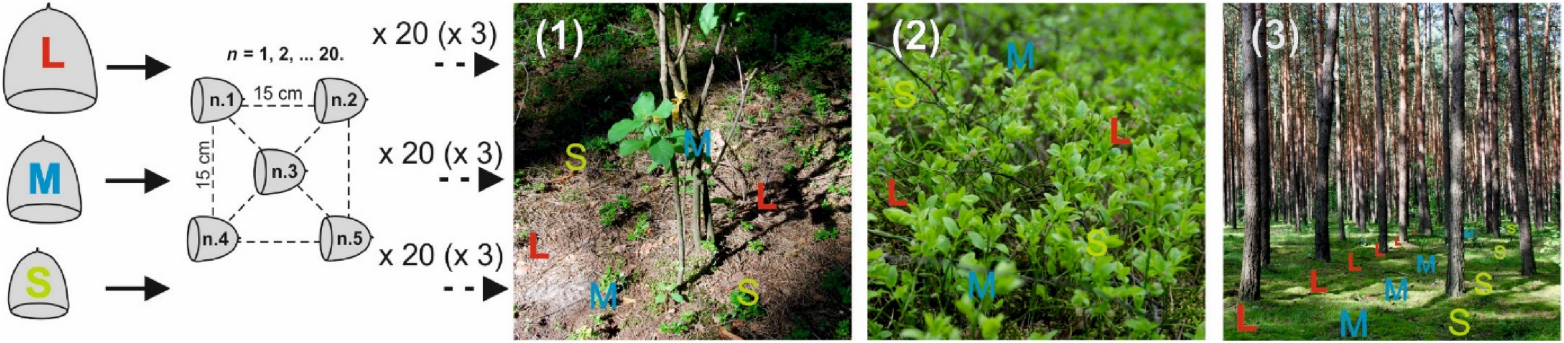
The scheme of distribution of large (L), medium (M), and small (S) acorns of *Quercus rubra* (300 acorns for each microsite studied), sown in five acorn sets (n.1, …, n.5) in the soil under shrubs (1), buried under the moss cover within clumps of bilberry (2), and in open areas (3), in the mesic Scots pine forest. Sets (× 20, in each of three types (× 3) of microsites studied) of large‐, medium‐, and small‐sized acorns were placed alternately at half‐meter intervals around shrub stem/stems and one‐meter intervals within bilberry clumps, and in the open areas, they were located in separate rows with one‐meter intervals. Their locations in the field were marked by ribbons with different colors for each of the acorn‐size classes.

To verify the results of the previous study (Woziwoda et al., 2018b), acorns were sown in three types of microsites: in the open space, within clumps of bilberry, and under shrubs of silver birch and alder buckthorn. To imitate the behavior of animals in seed hoarding, acorns were buried under the compact moss layer in the open space, under a loose moss layer hanging on dense shoots of bilberry, and under shrubs—buried in the soil at a depth of 4–5 cm. Acorns, 5 per set, were sown regularly in 20 subplots for each of the conditions studied (100 acorns × 3 size classes × 3 types of microsite) and consecutively numbered (Figure 1).

The location of plots in the forest space was marked permanently with a white number painted on the nearest Scots pine tree or on a wide ribbon tied to the trunk of a shrub, which allowed us to find every acorn/seedling in June and September, in the subsequent 3 sequential years. This also allowed us to avoid the inclusion in the study of other seedlings that germinated nearby from acorns dispersed in the meantime by birds or rodents. Unfortunately, during the summer of 2020, the shrub layer was cleared in the whole area of the Scots pine monoculture, not allowing us to collect September 2020 data for this microsite type.

### 2.3 Trait measurements

#### 2.3.1 Acorn survival and germination

Acorn survival and germination were checked for the first time in June 2018. In the case of a lack of a seedling or an invisible sprout, the top layer of the moss or soil was gently removed to determine whether the acorn was dead, stolen by post‐dispersal acorn‐consumers, or non‐germinated, and then acorns were re‐covered immediately, since non‐germinated acorns might germinate later. Acorns were classified as: (1) dead, when the acorn was non‐germinated and overgrown by black mycelium of fungi; (2) stolen, when the space was empty; (3) non‐germinated—for acorns without signs of destruction; (4) sprouting acorns—for acorns germinated to a leafless short sprout; and (5) successfully germinated and developed into a seedling with a leafy stem.

Sprouting acorns and seedlings were monitored to the end of the experiment, that is, to September 2020, except seedlings under shrubs which were monitored to June 2020. In the latter case, however, we assumed that seedlings noted in June 2020 could survive to September 2020, and they were classified for the last stage of the study as “seedlings (unsure).” During the experiment, mortality of sprouting acorns and seedlings, as well as seedling resprouting, that is, the development of a new leafy stem, were noted.

#### 2.3.2 Effects of microsite and acorn size on seedling growth and survival

Seedling survival and growth were inventoried twice a year in 2018, 2019 and 2020. The winter survival and spring growth were analyzed in June (VI), and the summer survival and growth were analyzed in September (IX) before the natural abscission of leaves in the autumn. Each time each seedling was measured for the stem height (=length) from the root collar—that is, from the transition zone between the above‐ and belowground portion to the stem top, and for the basal diameter (with the use of the caliper). The leaf number was counted, and the blade length and width of the biggest leaf were measured. The other characteristics of leaves were also considered, such as their damage by large herbivores (leaf blade eaten, only the leaf petiole remained), or by insects (i.e., perforation or skeletonization of the blade, and/or damage of the leaf margin), and infection by fungi (visible leaf discoloration). These variables were treated as Boolean (presence/absence).

The complete loss of leaves, that is, total seedling defoliation in the early (in June) or late (in September) summer, the decrease (partial defoliation) or increase of the leaf number during the summer, as well as the development of new leaves (noted in September) during summer on a stem that was leafless in June were also noted to analyze their effects on seedling growth and survival.

#### 2.3.3 Quality of 3‐year‐old seedlings (growing in the open space and within bilberry clumps)

After the last inventory in September 2020, all living seedlings were dug up with roots, cleared of soil, and collected for final measures. The length of the seedling root—from the root collar to the end of the longest root—, was measured before dividing the seedling into biomass components. Leaves, stems and roots were labeled and transported to the laboratory. Leaves were scanned and their total area for each seedling was measured using the Image_J program. Next, all biomass components were dried at 60°C for 24 h and weighed with an accuracy of 0.001 g to analyze the below‐ and aboveground accumulation of biomass.

### 2.4 Data analyses

We conducted all analyses using R software (R Core Team, 2023). We assessed acorn survival after germination using generalized linear mixed‐effects models (GLMMs) for successful germination, seed theft, and acorn death, assuming binomial distributions of dependent variables. In these models we used microsite and seed size as fixed effects and subplot as a random intercept. We presented results as both probabilities and odds ratios to show effect sizes. The odds ratio is a quotient of event probabilities between two groups of interest, showing how much higher/lower the probability of a particular event is. For GLMMs, we used the *lme4* (Bates et al., 2015) and *lmerTest* (Kuznetsova et al., 2017) packages. To assess survival probability over the whole study period, we used the Cox proportional hazard model, accounting for seed size and microsite, implemented in the *survival* package (Therneau & Grambsch, 2000). We used linear mixed‐effect models (LMMs) to assess seedling growth characteristics (i.e., stem diameter, height, leaf width, area, and number) over study dates, microsites, and seed sizes, and accounting for leaf and stem damage categories. In such models, we used subplot as a random intercept. We also used LMMs to assess seedling characteristics at the end of the study (biomass, its allocation, root length, and leaf traits), accounting for seed size and microsite as fixed effects and subplot as a random intercept. For all LMMs, we selected best‐fit models using Akaike's information criterion, corrected for small sample size (AICc), and reported AICc of the null (intercept and random effects only) model (AICc_0_). We also provided conditional and marginal coefficients of determination (${R^{2}}_{c}$ and ${R^{2}}_{m}$, respectively). ${R^{2}}_{m}$ indicates the amount of variance explained by fixed effects only, while ${R^{2}}_{c}$ indicates the amount of variance explained by both random and fixed effects (Nakagawa & Schielzeth, 2013). These two coefficients and AICc were calculated using the *MuMIn* package (Bartoń, 2017). For models, we reported results using ANOVA design. We presented marginal effects (i.e., estimates assuming mean values of other predictors and excluding random effects) obtained using the *ggeffects* package (Lüdecke, 2018). While interpreting results, we followed the American Statisticians Association statement (Wasserstein & Lazar, 2016) and we relied on effect sizes rather than on *p*‐values only, as this latter statistic is highly sample size dependent and can lead to misinterpretation of some significant findings.

## 3 RESULTS

### 3.1 Acorn and seedling survival and development

**FIGURE 2:**
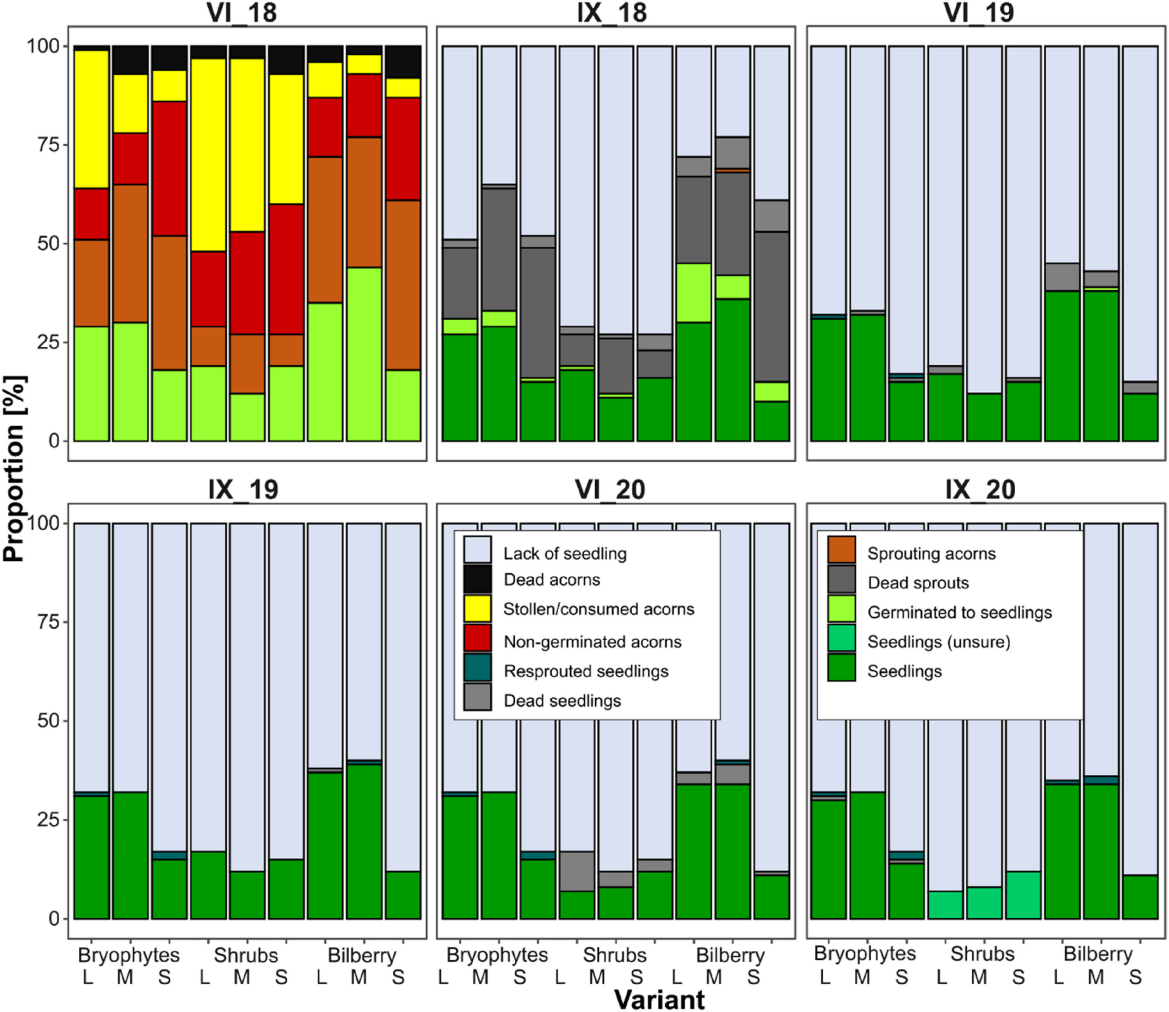
Overview of *Q. rubra* acorn and seedling survival and development in three types of microsites: in open areas with dense moss cover (bryophytes), under shrubs, and within bilberry clumps, during the whole period of study (seeds were sown in October 2017, and monitored in June and September for 3 years, i.e., until September 2020; dates are indicated above each panel). Acorns in three size classes: large (L), medium (M), and small (S), were buried under moss cover (in open areas and in bilberry) and in soil (under shrubs).

From the pool of 900 acorns sown in October 2017, in June 2018 we found 461 acorns (51.2%) successfully germinated: 237 of them were sprouted to leafless stems and 224 were developed to leafy seedlings. A total of 195 acorns (21.7%) were non‐germinated (and they all turned out to be non‐viable), 41 (4.6%) were dead due to fungi infection and 203 (22.6%) were not found (Figure 2). In total, almost half (48.8%) of acorns were lost after the first winter and spring. During the second control after summer (in September 2018), the total acorn losses increased to 74.2%, and at the end of the experiment (September 2020), they increased to 79.1%, that is, one‐fifth (20.9%) of sown acorns successfully developed into seedlings which survived to the end of the experiment. The final losses in the pool of sown acorns were caused by: acorn pilferage (28.5% of lost acorns), the lack of acorn germination (27.4%), the death of sprouting acorns (27.7%), leafy seedling death (10.7%), and the acorn death caused by contamination by fungi (5.8% of lost acorns).

A total of 82 leafy seedlings died during the experiment, their mortality was the highest at the end of the first year (41.5% of all dead seedlings were noted in September 2018), and then after the winter seasons of 2019 and 2020 (23.2%, and 31.7%, respectively). Sporadically, after the suspected death of a seedling, a new stem regrew (10 cases), and 60% of these resprouted seedlings survived to the end of experiment. Most (83.1%) of the acorns with delayed germination (noted as sprouting acorns in June, 2018) died during the experiment. The survival rate of seedlings that developed later (from acorns with delayed germination) was 13.1%, while the survival of leafy seedlings that were noted during the first control (in June 2018) was 70.1%.

Acorn and seedling survival differed both for acorns in different size classes sown within the same specific type of microsite, as well as for acorns from the same size class sown in different microsites (Figure 2). Among acorns noted as “sprouting” in June 2018, 9.9%, 12.1%, and 23.9% survived the summer under bryophytes in open areas, under shrubs, and in bilberry clumps, respectively. Overall, after the first year, 30%, 72.3%, and 44% of the acorns died in these microsites. After 3 years, 26.3%, 9%, and 27.3% of acorns sown in open areas, under shrubs, and in bilberry clumps survived as seedlings, respectively.

#### 3.1.1 Models of acorn survival

**FIGURE 3:**
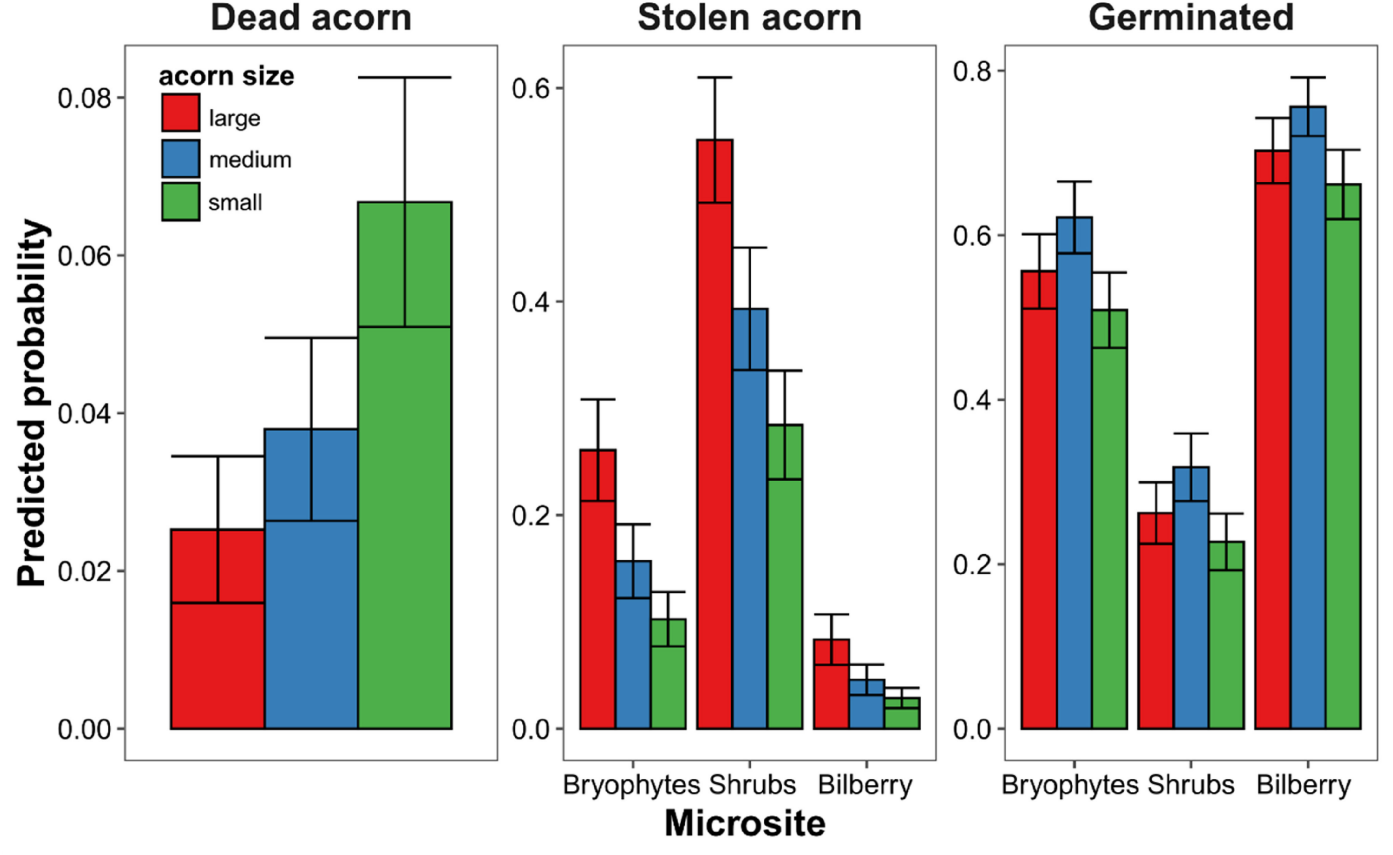
Marginal mean (+SE) probabilities of *Q. rubra* acorn death, pilferage, and germination among microsites (i.e., under dense moss cover (bryophytes) in open areas and soil under shrubs, and under a loose moss layer in bilberry) and acorn size categories (large, medium, and small), assessed using generalized linear mixed‐effects models.

The probability of successful germination differed among microsites and seed sizes, with the highest under bilberry and for medium‐size seeds (Figure 3; Table 1). The odds ratio between bilberry and shrub microsites was 1.895 ± 0.187, bilberry and bryophytes 0.635 ± 0.176, and between shrubs and bryophytes 1.260 ± 0.179. The odds ratio between medium and small acorn sizes was 0.461 ± 0.179, between medium and large 0.271 ± 0.179, and between large and small 0.190 ± 0.178. The probability of finding an empty space (places where the acorn was stolen) differed among microsites and seed sizes, with the highest under shrubs and for large seeds. The odds ratio between bilberry and shrub microsites was 2.600 ± 0.277, bilberry and bryophytes 1.350 ± 0.286, and between shrubs and bryophytes 1.250 ± 0.200. The odds ratio between medium and small acorn sizes was 0.488 ± 0.234, between medium and large 0.640 ± 0.212, and between large and small 1.128 ± 0.227. The probability of acorn death was low, and only seed size revealed differences higher than 0.01.

**TABLE 1:** Analysis of variance for probabilities of acorn death, pilferage, and germination among microsites and seed size categories, assessed using generalized linear mixed‐effects models (with acorn block as a random intercept).

Response	Variable	df	Sum of squares	Mean square	χ^2^	Pr (>χ^2^)	Random effect SD
Dead acorn	Microsite	2	0.049	0.024	0.052	0.974	0.421
${R^{2}}_{m}$ = .007, ${R^{2}}_{c}$ = .013	Acorn size	2	6.286	3.143	6.508	0.039	–
Stolen acorn	Microsite	2	89.268	44.634	99.842	<0.001	0.719
${R^{2}}_{m}$ = .177, ${R^{2}}_{c}$ = .245	Acorn size	2	25.841	12.921	25.575	<0.001	–
Germination	Microsite	2	106.151	53.075	106.543	<0.001	0.345
${R^{2}}_{m}$ = .136, ${R^{2}}_{c}$ = .172	Acorn size	2	6.748	3.374	6.677	0.035	–

**FIGURE 4:**
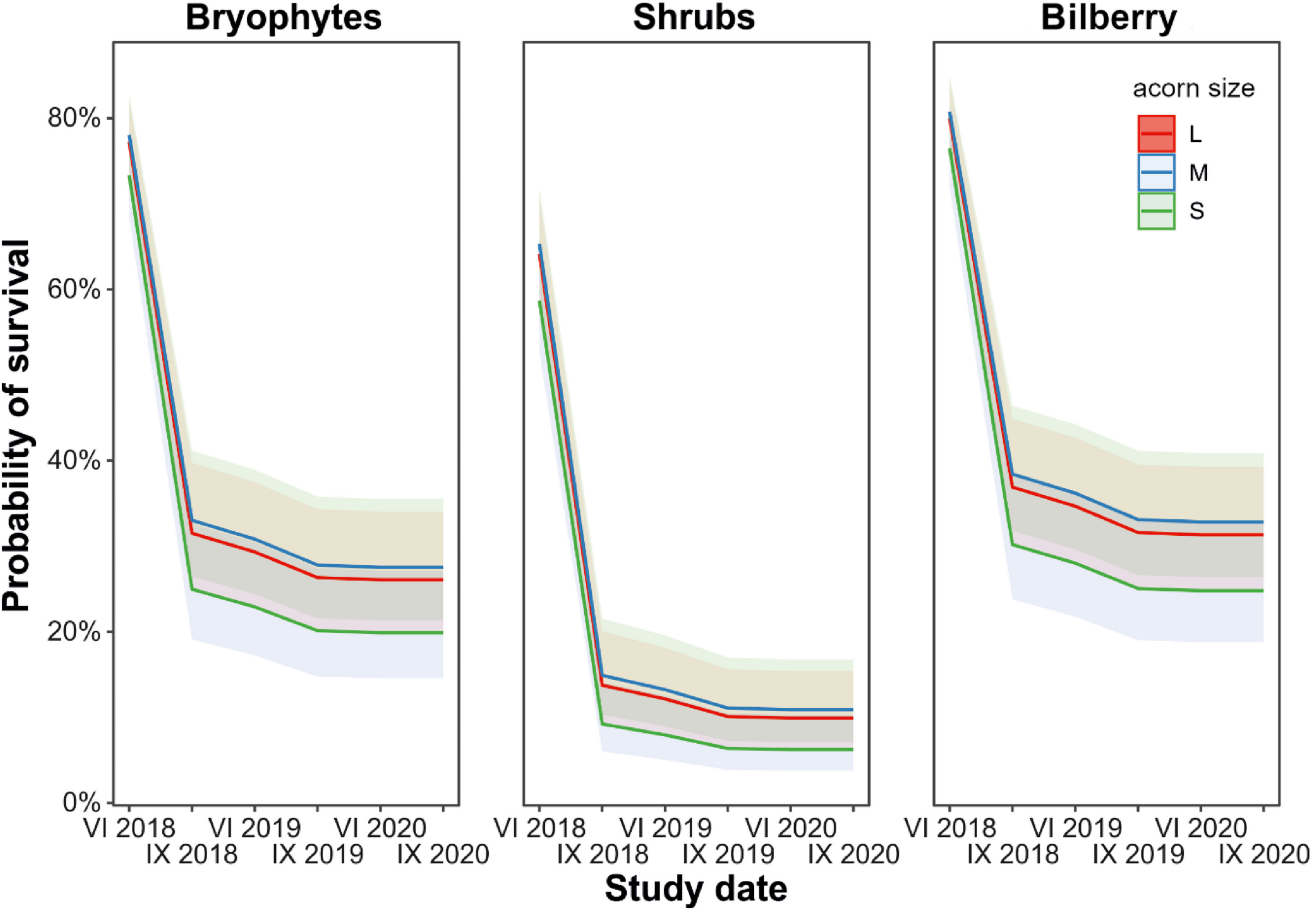
Mean (±SE) survival probability for sown acorns of *Q. rubra* across the whole study period and among acorn sizes (L—large, M—medium, and S—small) and microsites (i.e., under moss cover in open areas and in bilberry, and in soil under shrubs), assessed using Cox proportional hazard model (Table 2).

The best‐fit Cox proportional hazard model accounted for both microsite and seed size (AICc = 8789.4, AICc_0_ = 8853.0) and revealed that both factors significantly affected the survival of acorns (Figure 4; Table 2). Survival of large seeds after sowing was similar to medium seeds (0.77 and 0.78, respectively), but higher than that of small seeds (0.73), with similar differences at the end of the study (0.26, 0.28, and 0.20, respectively). Bryophytes and bilberry provided higher survival than shrubs both at the beginning (0.77, 0.80, and 0.64, respectively) and end of the study (0.26, 0.31, and 0.10, respectively).

**TABLE 2:** Cox proportional hazard model for acorns across the whole study period and among seed sizes and microsites (microsite = bryophytes and seed size = large were used as default levels).

Variable	Estimate	SE	*z*	*p*
microsite = shrubs	0.541	0.091	5.959	<.001
microsite = bilberry	−0.147	0.096	−1.537	.124
acorn size = medium	−0.041	0.094	−0.439	.660
acorn size = small	0.183	0.091	2.010	.044

### 3.2 Seedling growth

**FIGURE 5:**
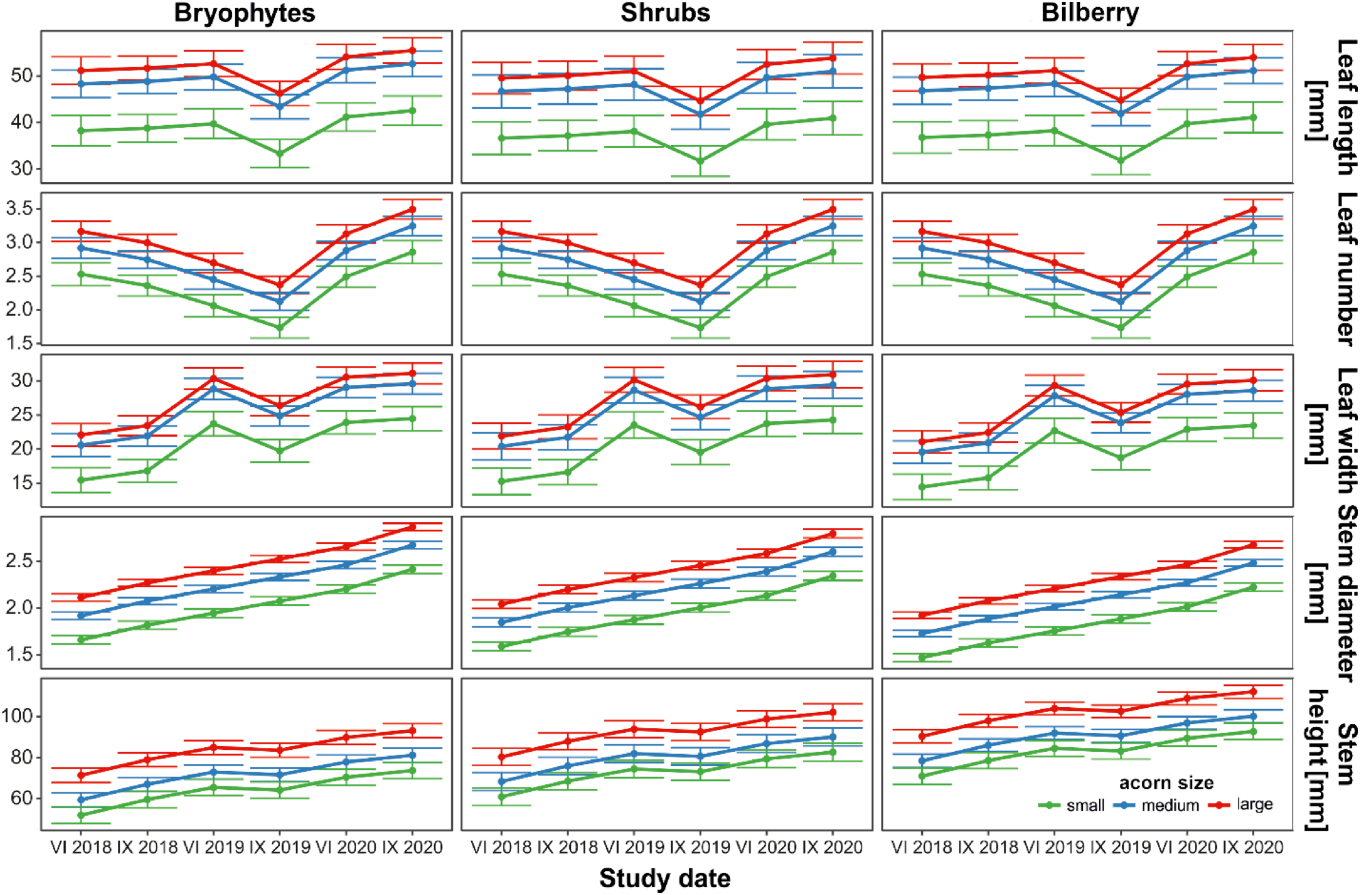
Marginal mean (±SE) of seedling characteristics among microsites and acorn size categories over study dates, assessed using linear mixed‐effects models (Table 3). The figure shows the dependence of the growth of seedlings in various microhabitats on the size of the acorns, convergence in time (parallel lines), and influenced by factors other than acorn size and type of microsite (upward trends of lines and line breaks; for example, partial defoliation of all seedlings recorded in September 2019 was the seedlings' reaction to the negative impact of the hot and dry summer of 2019).

The best model of stem height over time comprised all of the hypothesized predictors, and the leaf damage categories had neither statistically nor ecologically significant effects (Figure 5; Table 3). On average, stem damage decreased stem height by 13.7 ± 1.8 mm. Across study dates, we found the tallest seedlings in *Vaccinium* (average 92.2 ± 2.4 mm) while the lowest were in the open areas in bryophytes (73.2. ± 2.7 mm), and there was a significant advantage of large and medium acorns (average 93.0 ± 2.5 and 81.0 ± 2.6 mm, respectively) over small seeds (73.6 ± 3.2 mm). The model of stem diameter revealed the impact of three predictors—seed size, microsite, and study date. Across study dates, we found the highest diameter in the open sites in bryophytes while the lowest was in bilberry, however, the average difference was <0.2 mm. For seed size, we found a significant advantage of large and medium acorns (average seedling diameter amounted to 2.38 ± 0.03 and 2.19 ± 0.02 mm, respectively) over small seeds (1.93 ± 0.03 mm). The model of leaf number did not account for microsite effects, but accounted for study date, leaf damage categories, and seed size; the predicted leaf number dropped in all variants in Sept 2019. The predicted leaf number was higher for large and medium seeds (average 2.9 ± 0.1 and 2.7 ± 0.1 leaves, respectively) over small seeds (2.3 ± 0.1). Fungi and insects decreased leaf number on average by 0.5 ± 0.1 leaves, while herbivores by 1.3 ± 0.1. Models of leaf width and length revealed the same trends.

**TABLE 3:** Linear mixed‐effects models of seedling growth characteristics over study dates.

Response	Variable	Sum of squares	Mean square	Numerator df	Denominator df	*F*	*p*	Random effects SD
Height	Microsite	6573.6	3286.8	2	256.4	15.6	<.001	Acorn id
AICc = 10671.6	Acorn size	5583.4	2791.7	2	256.0	13.3	<.001	23.00
AICc_0_ = 10982.8	Leaf fungi	585.8	585.8	1	1082.7	2.8	.095	Residual
${R^{2}}_{m}$ = .220	Leaf herbivory	208.0	208.0	1	992.6	1.0	.320	14.49
${R^{2}}_{c}$ = .778	Leaf insects	183.9	183.9	1	1036.7	0.9	.350	
	Stem damage	25,450.9	25,450.9	1	990.4	121.2	<.001	
	Date	36,600.0	7320.0	5	985.3	34.8	<.001	
Diameter	Microsite	0.7	0.4	2	254.3	12.5	<.001	Acorn id
AICc = −161.1	Acorn size	3.0	1.5	2	256.8	50.7	<.001	0.26
AICc_0_ = 1035.3	Date	63.0	12.6	5	976.2	431.8	<.001	Residual
${R^{2}}_{m}$ = .495								0.17
${R^{2}}_{c}$ = .848								
Leaf number	Acorn size	31.4	15.7	2	244.7	9.1	<.001	Acorn id
AICc = 4388.6	Leaf fungi	22.8	22.8	1	1212.2	13.2	<.001	0.63
AICc_0_ = 4678.4	Leaf herbivory	214.2	214.2	1	1181.0	123.9	<.001	Residual
${R^{2}}_{m}$ = .222	Leaf insects	38.9	38.9	1	1188.4	22.5	<.001	1.32
${R^{2}}_{c}$ = .369	Date	137.1	27.4	5	1038.3	15.9	<.001	
Leaf length	Microsite	259.1	129.5	2	237.1	0.3	.773	Acorn id
AICc = 11304.1	Acorn size	12,018.5	6009.3	2	220.0	12.0	<.001	11.65
AICc_0_ = 11733.0	Leaf fungi	1129.7	1129.7	1	1212.2	2.2	.134	Residual
${R^{2}}_{m}$ = .271	Leaf herbivory	140,228.0	140,228.0	1	1106.4	279.1	<.001	22.41
${R^{2}}_{c}$ = .426	Leaf insects	2357.2	2357.2	1	1169.6	4.7	.031	
	Stem damage	7214.2	7214.2	1	1123.3	14.4	<.001	
	Date	9127.2	1825.4	5	1013.6	3.6	.003	
Leaf width	Microsite	102.5	51.2	2	251.0	0.3	.710	Acorn id
AICc = 9852.8	Acorn size	2922.1	1461.1	2	235.0	9.8	<.001	6.81
AICc_0_ = 10270.3	Leaf fungi	243.5	243.5	1	1213.9	1.6	.202	Residual
${R^{2}}_{m}$ = .268	Leaf herbivory	39,863.4	39,863.4	1	1104.0	266.4	<.001	12.23
${R^{2}}_{c}$ = .441	Leaf insects	684.5	684.5	1	1165.4	4.6	.033	
	Stem damage	3756.3	3756.3	1	1118.7	25.1	<.001	
	Date	12,547.6	2509.5	5	1023.1	16.8	<.001	

*Note*: For model selection, see Table S1.

### 3.3 Seedling characteristics

**FIGURE 6:**
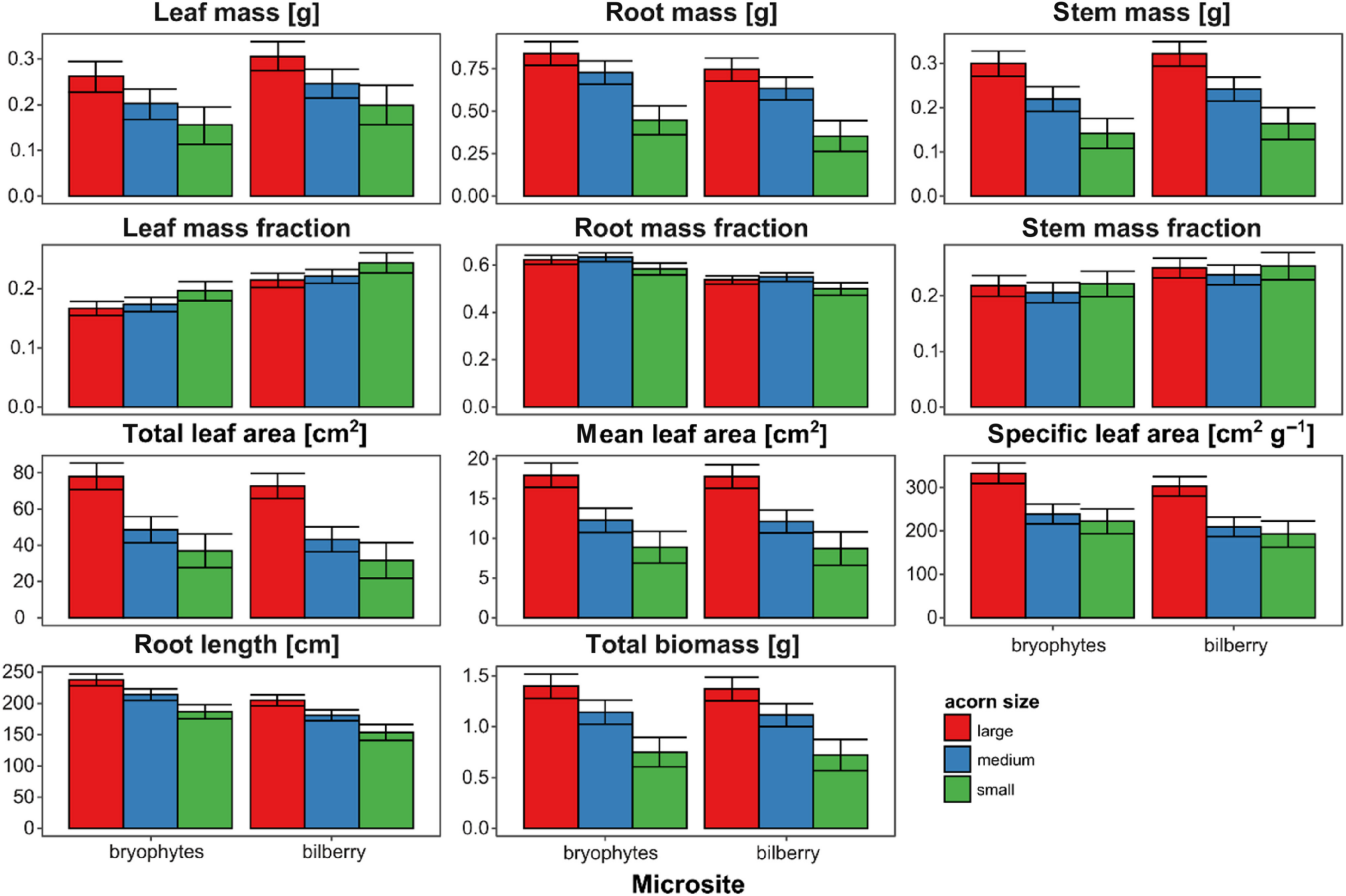
Characteristics of 3‐year‐old *Q. rubra* seedlings, growing from large, medium, and small acorns sown under moss cover in the open area (bryophytes) and bilberry clumps. Marginal mean (+SE) seedling characteristics among microsites and acorn size categories were assessed using linear mixed‐effects models (Table 4).

A comparison of seedling characteristics at the end of the study revealed that those originating from large acorns had higher root and stem mass, total, mean, and specific leaf area, root length, and total biomass (Figure 6; Table 4). We found the highest effect sizes for leaf characteristics and total biomass, where the biomass of seedlings from large acorns was twice that of small acorns. However, biomass allocation was lower in leaves (0.166 ± 0.012 vs. 0.196 ± 0.017 in bryophytes and 0.214 ± 0.011 vs. 0.243 ± 0.017 in bilberry) and higher in roots (0.622 ± 0.018 vs. 0.584 ± 0.025 in bryophytes and 0.537 ± 0.018 vs. 0.499 ± 0.026 in bilberry), and it was similar to seedlings originating from medium acorns. For microsites, we observed differences in root length and biomass allocation into leaves and roots: seedlings growing in bryophytes had longer roots and allocated more biomass into roots than into leaves.

**TABLE 4:** Linear mixed‐effects models of seedling characteristics over study dates.

Response	Variable	Sum of squares	Mean square	Numerator df	Denominator df	*F*	*p*	*R* ^2^		Random effects	SD
Leaf mass	Microsite	0.033	0.033	1	77.449	1.533	.219	${R^{2}}_{m}$	.060	Acorns block	0.111
	Acorn size	0.118	0.059	2	83.022	2.769	.069	${R^{2}}_{c}$	.403	Residual	0.146
Root mass	Microsite	0.145	0.145	1	76.073	1.623	.207	${R^{2}}_{m}$	.117	Acorns block	0.241
	Acorn size	1.401	0.701	2	81.723	7.842	.001	${R^{2}}_{c}$	.465	Residual	0.299
Stem mass	Microsite	0.006	0.006	1	81.132	0.567	.454	${R^{2}}_{m}$	.137	Acorns block	0.110
	Acorn size	0.161	0.081	2	84.689	8.217	.001	${R^{2}}_{c}$	.612	Residual	0.099
Leaf mass fraction	Microsite	0.088	0.088	1	153.000	13.770	<.001	${R^{2}}_{m}$	.090	Acorns block	0.000
	Acorn size	0.017	0.008	2	153.000	1.294	.277	${R^{2}}_{c}$	.090	Residual	0.080
Root mass fraction	Microsite	0.260	0.260	1	55.963	18.127	<.001	${R^{2}}_{m}$	.117	Acorns block	0.024
	Acorn size	0.044	0.022	2	69.630	1.553	.219	${R^{2}}_{c}$	.151	Residual	0.120
Stem mass fraction	Microsite	0.017	0.017	1	60.516	2.711	.105	${R^{2}}_{m}$	.027	Acorns block	0.064
	Acorn size	0.003	0.002	2	66.049	0.244	.784	${R^{2}}_{c}$	.404	Residual	0.080
Total leaf area	Microsite	793.503	793.503	1	74.391	0.477	.492	${R^{2}}_{m}$	.127	Acorns block	16.597
	Acorn size	32,226.962	16,113.481	2	84.535	9.695	<.001	${R^{2}}_{c}$	.251	Residual	40.767
Mean leaf area	Microsite	0.737	0.737	1	86.045	0.009	.926	${R^{2}}_{m}$	.117	Acorns block	2.659
	Acorn size	1613.010	806.505	2	97.644	9.560	<.001	${R^{2}}_{c}$	.186	Residual	9.185
Specific leaf area	Microsite	15,648.817	15,648.817	1	84.655	1.471	.229	${R^{2}}_{m}$	.133	Acorns block	77.587
	Acorn size	171,594.322	85,797.161	2	90.174	8.064	.001	${R^{2}}_{c}$	.446	Residual	103.146
Root length	Microsite	22,863.573	22,863.573	1	70.646	11.519	.001	${R^{2}}_{m}$	.167	Acorns block	28.716
	Acorn size	29,877.254	14,938.627	2	78.006	7.526	.001	${R^{2}}_{c}$	.411	Residual	44.552
Total biomass	Microsite	0.010	0.010	1	79.257	0.050	.824	${R^{2}}_{m}$	.114	Acorns block	0.451
	Acorn size	2.933	1.467	2	83.292	7.517	.001	${R^{2}}_{c}$	.566	Residual	0.442

## 4 DISCUSSION

Our experimental study revealed that the survival and development of *Q. rubra* acorns and seedlings in the mesic Scots pine forest depended largely on acorn size (i.e., large, medium, and small) and differed among microsites studied (i.e., in open areas and bilberry, and under shrubs).

The majority of sown acorns did not survive. However, the overall survival rate was twice as high as that reported by Dyderski and Jagodziński (2019), who found an average survival of 12.5 ± 2.2% (after 1 year) in various types of temperate forests. The difference can result from the fact that the behavior of acorn hoarders, that is, active searching for acorns formerly cached and their consumption (Wang et al., 2014, and references therein), was excluded in our study (otherwise the final losses could have been even higher). On the other hand, acorns for sowing were randomly selected, while animals choose seeds for hoarding (Pesendorfer et al., 2016), and some rodents and birds prefer larger acorns (rich in food reserves) and are less likely to eat them in situ than to remove and hoard them (Forget et al., 1998; Vander Wall, 2001; Xiao et al., 2006), while others prefer smaller acorns as they are easier to carry (Muñoz et al., 2012; Muñoz & Bonal, 2008). In the first scenario, larger acorns would dominate among those dispersed outside the area of cultivation which facilitates higher acorn survival and seedling establishment and recruitment. In the second scenario, preferences for small acorns would result in northern red oak regeneration failure; as we showed in this study, a majority of small seeds are lost (Figure 3). However, the use of acorns by animals is very complex and context dependent, and relies, among others, on the composition and size of animal guilds in a specific forest area, crop abundance of acorns in a specific year, or availability of other food resources (Wróbel et al., 2022). Nevertheless, this study revealed that the very large losses in the pool of sown acorns were caused by acorn pilferage, the lack of acorn germination, and the death of sprouting acorns.

### 4.1 Non‐germinated acorns

Acorns for sowing were randomly selected and their viability was assessed only visually (not tested), thus some of them were naturally unable to germinate (De Groote et al., 2018); however, the high “initial” non‐viability of acorns produced by *Q. rubra* can significantly impact its regeneration. The noted proportion of non‐germinated acorns was relatively high, as it constituted over one‐fifth of the total pool of sown acorns (Figure 2). The proportion of non‐viable seeds in the seed crop produced by the adjacent northern red oak trees, however, can vary significantly in sequential years, for example, in 2018, it amounted to only 7%, while in 2019, it was 75% (Woziwoda et al., 2023).

The lack of acorn germination could also result from seed destruction after sowing, for example, due to seed desiccation (Goodman et al., 2005) or over‐freezing, post‐dispersal predation by insects or infection by fungi (Dey & Parker, 1996). Among ungerminated acorns with visible signs of fungal pathogens, the highest mortality rate was noted for the small‐sized acorns (Figures 2 and 3), indicating that they are the most susceptible to fungal infection. We suppose that all acorns could have been at risk of fungal infection even before sowing, as numerous acorns overgrown with black mycelium were found in the litter under the *Q. rubra* canopy (personal observations). However, acorns could also be infected after sowing (Washington, 2003; and references therein), and it is likely that the high humidity among dense bilberry shoots most favored the development of pathogenic fungi. To confirm that observation, we encourage further, specialized myco‐ecological studies.

### 4.2 Acorn pilferage

High acorn losses due to acorn theft could be partially explained by the long‐time exposure of seeds sown in the fall season to predators (Birkedal et al., 2010; Bonner, 2008). In the native range of *Q. rubra*, post‐dispersal acorn predation is indicated as one of the most important factors decreasing the natural regeneration of northern red oak populations (Crow, 1992; Dey & Parker, 1996). However, the post‐dispersal acorn losses due to acorn theft are context dependent (Bartlow et al., 2018; Buckley et al., 2006; González‐Rodríguez & Villar, 2012; Lichti et al., 2017; Löf et al., 2019) and differ significantly among sites (Birkedal et al., 2009, 2010; Buckley et al., 1998; Martelletti et al., 2018; Pèrez‐Ramos & Marañón, 2008; Schupp et al., 2019; Smallwood et al., 2001). The highest rates of acorn disappearance were noted under shrubs (Figures 2 and 3); however, this was surprising because in previous studies the highest frequency of *Q. rubra* juveniles was recorded under shrubs (Woziwoda et al., 2018b).

This study revealed that acorn pilferage was positively correlated with acorn size, regardless of the type of microsite (Figure 3), likely because larger acorns are more easily found by acorn consumers/pilferers (Vander Wall, 1990). They smell more intensively (Luft et al., 1994), and this factor could be responsible for the high losses of acorns sown in the open space. In few cases, acorns were stolen in mass and the range of theft covered a few adjacent subplots, with the highest number of 28 large acorns stolen from six subplots located close to each other. This indicates a concentration of searches by acorn pilferers after finding a single acorn and confirms the assumption that pilferers are more likely to find and pilfer seeds that are closer together (Stapanian & Smith, 1984; Vander Wall, 1990). Searching for acorns among dense bilberry stems (Woziwoda, Dyderski, & Jagodziński, 2019) was more difficult, thus they were least often pilfered. The changeable weather in autumn 2017 and winter 2017/2018 (Figure S1) surely enhanced the activity of rodents searching for food (Orrock & Danielson, 2009; Pucek et al., 1993), which could increase acorn losses (Pereira & Koprowski, 2019).

### 4.3 Acorn and seedling survival and seedling growth

*Q. rubra* seeds and seedlings are highly susceptible to soil/air temperature and moisture (Crow, 1988; Dey & Parker, 1996; Noland et al., 2013; Suszka & Krawiarz, 1971; Suszka & Tylkowski, 1981), thus some acorns deposited under shrubs (in the bare soil) could die due to extremely low temperatures noted in winter 2018 (Figure S1). Acorn burial under a dense moss cover was protective for most of the northern red oak acorns (Figure 2), and this was especially important for large seeds, which are characterized by rapid germination (Ganatsas & Tsakaldimi, 2013; Goodman et al., 2005; Pritchard, 1991; Ramírez‐Valiente et al., 2009). An interesting finding was that small acorns buried in the moss remained in the dormancy stage longer and sprouted later—in the early summer (Figure 2). Delayed germination and slow seedling growth allow avoidance of the negative impacts of spring frosts (Aizen & Woodcock, 1996), however, long acorn dormancy and rapid seedling development in hot and dry summer days could be responsible for the extremely high mortality of germinating seeds and early seedlings (Figure 2). Paradoxically, protection of small acorns from frost by moss cover could be too good, as the stratification requirements of *Q. rubra* seeds may not be completely satisfied (Bonner, 1973), but further study is necessary. However, neither the shade of bilberries or shrubs nor the moss cover sufficiently protected the rising sprouts and seedlings from the summer drought (Bonner, 2008; Walters et al., 2023; Weber & Gates, 1990). Admittedly seedlings can compensate for losses connected with foliage reduction by delayed autumnal leaf senescence together with a delayed bud burst in the subsequent spring; however, it has to be correlated with the re‐watering of soil after summer drought (Vander Mijnsbrugge et al., 2016) and with the lack of early autumnal frosts, and such conditions did not occur in the area studied (Figure S1). The most effective drought avoidance mechanism of oak seedlings, that is, reduction of the leaf area (Jacobs et al., 2009; Wright et al., 2004), however, was observed in our experiment and clearly noticed for all seedlings—regardless of acorn size category and type of microsite, in September 2019 (Figure 5), that is, after a dry spring and extremely dry and hot summer (Figure S1). The rate of height growth of shoots also slowed down then, while the growth in thickness of shoots was undisturbed, which indicated the allocation of resources into more permanent parts of the seedlings (Jacobs et al., 2009).

The positive correlations revealed between acorn size and acorn germination and survival percentage of seedlings were also described in the *Q. rubra* native range (Kormanik, Sung, Kormanik, et al., 1998; but see Auchmoody et al., 1994). Oaks growing from large acorns were taller and more vital than those growing from small acorns, however, differences between specimens from large and medium acorns were not significant. It suggests the presence of an acorn mass and size threshold above which *Q. rubra* seedlings develop successfully.

Delayed acorn germination meant a longer duration of leaf emergence and consequently shorter leaf longevity, that is, limited time for effective photosynthesis for seedling growth, especially important for early seedling survival (De Groote et al., 2018; McGraw et al., 1990). Seedlings growing from small acorns unfolded their leaves over the summer (up to September 2018), while those growing from larger acorns completed their leaf production in spring (before June 2018). According to Seiwa and Kikuzawa (1996), the simultaneous expansion and development of leaves at the beginning of the vegetative season allows the reduction of environmental stress and decreases the mortality rate after seedling emergence. However, the fast depletion of nutrient resources insufficient for initial root growth and the development of aboveground biomass to sustain the individuals via photosynthesis resulted in the highest mortality of seedlings growing from small acorns (De Groote et al., 2018). The noted intraspecific differences in seedling dimensions resulted from acorn size (Figure 6), but they were also modified by the environmental conditions of microsites (Koch et al., 2004; Long & Jones, 1996; Tozer et al., 2015). Despite relatively homogeneous light conditions prevailing under the Scots pine canopy, oak seedlings growing within bilberry clumps were protected at the initial phase of growth from direct sunlight by relatively tall bilberry stems (Woziwoda, Dyderski, & Jagodziński, 2019), and light deficiency likely forced the development of bigger leaves (Dyderski & Jagodziński, 2019; Kuehne et al., 2014). It also resulted in larger seedling dimensions noted in bilberry than in the other two types of microsites studied. Seedlings competing with bilberry for light grew taller, but their diameter was smaller, while seedlings growing in the open area, without the competition of herbaceous plants, invested more resources in roots and stem diameter, compared to the height. Results were in line with other studies showing positive relationships between oak regeneration biomass and understory species richness and diversity (Kolb & Steiner, 1990; Jensen et al., 2011; Dyderski & Jagodziński, 2018; but see Löf et al., 2021). The differences found, however, could also result from different forest soil fertility of forest site potential (De Groote et al., 2018).

### 4.4 Seedling damages—causes and consequences for their growth and survival

Our study revealed that the growth and survival of northern red oak seedlings in the studied mesic Scots pine forest were affected by the herbivory of large ungulates (Table 3). Admittedly browsing on small (<20 cm tall) oak seedlings is reported as low and classified as a minor mortality factor (Götmark et al., 2005); however, the pressure of ungulates on oak juveniles is context dependent (Kern et al., 2012; Löf et al., 2021), and in some cases, it can be very heavy (Averill et al., 2016; Dey & Parker, 1996; Granger et al., 2018; Riepšas & Straigyté, 2008). In a mosaic of forest patches with different tree species composition, like the studied Scots pine monoculture with open canopy and well‐developed herb and moss layers (where experimental plots were located, and where animals feed) and Scots pine‐northern red oak stands with closed canopies and completely reduced herbaceous and moss layers (where animals rest in shade), the migration of large herbivores like red deer *Cervus elaphus* L. and European roe deer *Capreolus capreolus* L. among stands is inevitable. The co‐occurrence of bilberry clumps and numerous seedlings and saplings of the northern red and pedunculate oaks (Woziwoda et al., 2018b) attract ungulates, especially in June–July, when they readily feed on bilberry leaves and fruits (Melis et al., 2006; Nestby et al., 2011), and occasionally on juvenile oaks. The browsed leafless seedlings were found both concentrated in one space and as dispersed single individuals growing within the same subplot among (1–4) other leafy specimens, which resulted from a different browsing strategy of herbivores. Some of them can forage leisurely in one place, which results in local damage to a higher number of seedlings, while others gnaw on seedlings when they wander (Cushman et al., 2020; Götmark et al., 2005; Oswalt et al., 2006, and references therein).

Seedlings that lost all of their photosynthetic capacity in June, however, usually recovered from defoliation before September. In the first year of growth, seedling recovery could depend on the availability of still unused resources stored in the cotyledons (although Aizen and Woodcock (1996) found no relationship between acorn size and survival of defoliated *Q. rubra* seedlings). In the subsequent years, the regrowth of defoliated/damaged seedlings was conditioned by resources deposited in roots, where a large proportion of carbohydrates assimilated during the previous vegetative season is stored, and translocated aboveground, for example, to the leaf buds, if necessary (Farmer, 1975; Jacobs et al., 2009; Wang et al., 2023). Isebrands and Dickson  (1994) and Jacobs et al. (2009) indicated that enlarging the root system (high root‐to‐shoot ratio) is a natural preparation of northern red oak seedlings for eventual release, as their shoots are often damaged or dieback, and resprout from adventitious buds at the root collar. However, resprouted seedlings as well as those that recovered from defoliation were shorter than undamaged ones, and 30% of them died in the next season. Their weak growth and survival, similar to seedlings growing from acorns with delayed germination, could be explained in part by shorter leaf longevity, insufficient photosynthetic capacity, and productivity that was too low during the vegetative season (Larson, 2011; Wang et al., 2023).

### 4.5 Verification of the previous results and hypothesis on “burial” and “nurse” effects on *Q. rubra* regeneration

Our study showed that acorns buried under the compact moss wefts more often developed into seedlings (Figure 2), so the positive “burial effect” was confirmed. Obtained results undermined the theory previously accepted by us (Woziwoda et al., 2018b) on the “nurse effect” of native shrubs on northern red oak seed germination and seedling growth. Rather, shrubs were much more frequently chosen by acorn hoarders as recognizable orientation points in the pine monoculture (Pesendorfer et al., 2016; Smallwood et al., 2001). However, for the same reason, acorns deposited under shrubs were more frequently found by acorn pilferers and consumed (or re‐cached) (Figures 2 and 3), and finally, an extremely small proportion of acorns deposited in this type of microsite developed into seedlings. Higher theft of acorns cached beneath the tree canopy than in open areas, as well as successful recruitment of acorns cached in the latter, neither pilfered nor recovered by granivorous rodents, was also shown by Muñoz and Bonal (2011) for holm oak *Q. ilex* in a savanna‐like landscape. The high number of *Q. rubra* juveniles growing under native shrubs could be explained by the lesser pressure of ungulates on the few surviving northern red oak seedlings each year, as large ungulates are more interested in foraging in bilberry and open areas, or on taller plants if they are available (González‐Rodríguez & Villar, 2012; Jensen et al., 2012), and in this context, the “nurse effect” of shrubs for *Q. rubra* seedlings could be confirmed.

In our previous study, we stated that seedlings growing under deciduous shrubs were favored by higher soil moisture (covered by the litter) and higher soil fertility resulting from the yearly supply of carbon and other elements from decomposing litter (Woziwoda et al., 2018b), but the 3‐year observations and study results contradict this. The leaves under shrubs were blown away by the wind, and the soil was bare or covered with a very thin layer of pine needles, thus it was not protective or favorable for acorns or seedlings (Bonner, 2008; Collins & Good, 1987; Facelli & Pickett, 1991; Launiainen et al., 2022). A different situation was found within dense clumps of bilberry, where each year soil humus was supplied with leaves, rich in macro‐ and micronutrients (Woziwoda, Dyderski, & Jagodziński, 2019). Admittedly *Q. rubra* is considered a stress‐tolerant tree species, able to survive highly reduced soil fertility (Chmura, 2014; Miltner & Kupka, 2016; Zerbe & Wirth, 2006); however, the occurrence of deciduous leaf litter and yearly supplies of nutrients favors its growth (Major et al., 2013; Woziwoda, Dyderski, Kobus, et al., 2019). Additionally, after rainfall, the moss layer occurring among bilberry stems holds higher humidity for a longer time, while in the more open areas, the moss “carpet” dries out more quickly (personal observations). The higher moisture of the first microhabitat could increase the maintenance of seed viability through the dormancy period (García et al., 2002; Kang et al., 2023) and explains the higher survival rate of northern red oak seedlings (Dickson & Tomlinson, 1996).

Limited nutrient and water availability is characteristic of soils of coniferous forests in general (Buczko et al., 2005; Zwydak et al., 2011), and their concentration in the near‐surface layer of the soil forces belowground competition of plants (Facelli & Pickett, 1991; Konôpka et al., 2005; Luo et al., 2023; Schuler & Robison, 2010). Digging seedlings for biomass research (in September 2020), we revealed a highly compacted tangle of plant roots, mainly of pines (*P. sylvestris* produces not only a long taproot but also a wide network of shallow lateral roots, Roberts, 1976; Čermák et al., 2008). The primary taproots of northern red oak seedlings were often twisted, indicating difficulties in “breaking through” this layer. This belowground pine–oak competition for nutrients and water could also limit the aboveground growth of oaks (Wang et al., 2023). As commercial forests worldwide are currently dominated by pine tree species (Richardson, 2000), the interspecific pine–oak belowground competition could be one of the key factors limiting natural regeneration of *Quercus* species (Fei et al., 2011; Lorimer, 1992; Luo et al., 2023). The successful artificial regeneration of *Q. rubra* in European forests (Nicolescu et al., 2018) is facilitated by limiting competition from other plants by mechanical scarification of the soil, that is, periodic destruction of the existing root layer (Miltner & Kupka, 2016). In nature, large areas with disturbed and uprooted soil are left by wild boars, *Sus scrofa* L., after their active plowing in the ground in search of food (Sütő et al., 2019). Wild boar occurrence in forest ecosystems may be an important factor favoring oak regeneration, albeit the longer presence of big ungulates limits the activity of acorn hoarders in open areas, and hidden acorns are actively searched out and consumed by boars (Focardi et al., 2000; Muñoz & Bonal, 2007; Suselbeek et al., 2014).

## 5 CONCLUSIONS

Our study shows that only a small percentage of *Q. rubra* acorns sown in the mesic Scots pine forest successfully develop into viable seedlings. Acorn and seedling survival and seedling growth are positively correlated with the acorn size, so the older *Q. rubra* stands or the more fertile forest sites where they occur, and where larger acorns are produced (Gręda et al., 2022), the higher the probability of successful *Q. rubra* regeneration (despite the highest pilferage of large acorns revealed here). The fast acorn germination in the spring and development of leafy seedlings in the first vegetative season are crucial for seedling survival, while longer acorn dormancy and their delayed germination in summer are what contribute to the greatest losses of seeds within bilberry clumps and in the open spaces, which also indicates negative consequences of the “too good protection” of seed burial. However, results confirmed the positive effects of acorn burial under the moss layer both on acorn survival and germination and seedling development. Success in seedling survival was three times higher within bilberry clumps and in open areas than under shrubs, but seedlings growing in these first two microsites were more often damaged by large ungulates, than those growing under shrubs. Seedlings “nursed” by shrubs survive and grow as saplings, and this explains the highest number of *Q. rubra* juveniles previously noted in this type of microsite. Therefore, to limit *Q. rubra* spread in the mesic Scots pine forest (if necessary), it is recommended to remove the shrub layer.

## AUTHOR CONTRIBUTIONS

**Beata Woziwoda:** Conceptualization (lead); data curation (equal); formal analysis (supporting); investigation (lead); methodology (equal); visualization (equal); writing – original draft (lead); writing – review and editing (lead). **Marcin K. Dyderski:** Formal analysis (lead); visualization (lead); writing – original draft (equal); writing – review and editing (equal). **Anastazja Gręda:** Conceptualization (equal); data curation (equal); investigation (equal); writing – original draft (supporting). **Lee E. Frelich:** Writing – original draft (equal); writing – review and editing (equal).

## FUNDING INFORMATION

This research did not receive any specific grant from funding agencies in the public, commercial, or not‐for‐profit sectors.

## CONFLICT OF INTEREST STATEMENT

The authors declare that they have no known competing financial interests or personal relationships that could have appeared to influence the work reported in this paper.

## Supporting information


Figure S1

Table S1

## Data Availability

Data are available as supplementary materials.
