# Supplementary material for: Verified hypotheses on the “nurse” and “burial” effects on introduced Quercus rubra regeneration in a mesic Scots pine forest
Source: Ecol Evol. 2024 Apr 1;14(4):e11185. doi: 10.1002/ece3.11185 (PMC10985384; doi:10.1002/ece3.11185)
Supplement: Supplementary file 1 — Figure S1 [file ECE3-14-e11185-s001.docx]

**Verified hypotheses on the ‘nurse’ and ‘burial’ effects on introduced *Quercus rubra* regeneration in a mesic Scots pine forest**

Beata Woziwoda*, Marcin K. Dyderski, Anastazja Gręda, Lee E. Frelich

*Corresponding author: Beata Woziwoda, University of Lodz, Faculty of Biology and Environmental Protection, Department of Geobotany and Plant Ecology, Banacha 12/16, 90-237 Łódź, Poland, [beata.woziwoda@biol.uni.lodz.pl](mailto:beata.woziwoda@biol.uni.lodz.pl)

**Figure S1.** Weather conditions (selected parameters) from 01/10/2017 to 30/09/2020.

Based on data of The Institute of Meteorology and Water Management – National Research Institute, Poland, presented on <https://meteomodel.pl/dane/historyczne-dane-pomiarowe/>(accessed 16 July 2023).

*We chose the minimum temperature at the ground level as this parameter is a good characteristic (better than a minimum daily temperature) of the environmental conditions on the forest floor, where acorns germinate and seedlings grow. This temperature was measured 5 cm above the ground, while the other air temperatures were measured at a height of 2 m above the ground (in shade).
