# Supplementary material for: Verified hypotheses on the “nurse” and “burial” effects on introduced Quercus rubra regeneration in a mesic Scots pine forest
Source: Ecol Evol. 2024 Apr 1;14(4):e11185. doi: 10.1002/ece3.11185 (PMC10985384; doi:10.1002/ece3.11185)
Supplement: Supplementary file 2 — Table S1 [file ECE3-14-e11185-s002.docx]

**Verified hypotheses on the ‘nurse’ and ‘burial’ effects on introduced *Quercus rubra* regeneration in a mesic Scots pine forest**

Beata Woziwoda^*^, Marcin K. Dyderski, Anastazja Gręda, Lee E. Frelich

*Corresponding author: Beata Woziwoda, University of Lodz, Faculty of Biology and Environmental Protection, Department of Geobotany and Plant Ecology, Banacha 12/16, 90-237 Łódź, Poland, [beata.woziwoda@biol.uni.lodz.pl](mailto:beata.woziwoda@biol.uni.lodz.pl)

**Table S1.** Results of model selection using dredge() function for linear mixed-effects models of seedling growth characteristics over study dates

| **Response** | **(Intercept)** | **Date** | **Fungi (0/1)** | **Herbivory (0/1)** | **Insects (0/1)** | **Microsite** | **Acorn size** | **Stem damage (0/1)** | **df** | **logLik** | **AICc** | **delta AICc** | **Akaike's weight** |
| --- | --- | --- | --- | --- | --- | --- | --- | --- | --- | --- | --- | --- | --- |
| Height | 78.04 | + | + | + | + | + | + | + | 16 | -5319.596 | 10671.6 | 0 | 0.351 |
| Height | 78.13 | + | + | + |  | + | + | + | 15 | -5321.158 | 10672.7 | 1.07 | 0.205 |
| Height | 78.24 | + | + |  | + | + | + | + | 15 | -5321.358 | 10673.1 | 1.47 | 0.168 |
| Height | 78.28 | + | + |  |  | + | **+** | **+** | **14** | **-5322.71** | **10673.8** | **2.13** | **0.121** |
| Height | 77.91 | + |  | + | + | + | + | + | 15 | -5322.18 | 10674.8 | 3.12 | 0.074 |
| Height | 78.01 | + |  | + |  | + | + | + | 14 | -5323.87 | 10676.1 | 4.45 | 0.038 |
| Height | 78.15 | + |  |  | + | + | + | + | 14 | -5324.246 | 10676.8 | 5.2 | 0.026 |
| Height | 78.19 | + |  |  |  | + | + | + | 13 | -5325.658 | 10677.6 | 5.98 | 0.018 |
| Height | 68.91 | + | + | + | + | + |  | + | 14 | -5336.752 | 10701.9 | 30.21 | 0 |
| Height | 69 | + | + | + |  | + |  | + | 13 | -5338.259 | 10702.8 | 31.18 | 0 |
| Height | 69.09 | + | + |  | + | + |  | + | 13 | -5338.563 | 10703.4 | 31.78 | 0 |
| Height | 69.14 | + | + |  |  | + |  | + | 12 | -5339.867 | 10704 | 32.35 | 0 |
| Height | 68.75 | + |  | + | + | + |  | + | 13 | -5339.415 | 10705.1 | 33.49 | 0 |
| Height | 88.95 | + | + | + | + |  | + | + | 14 | -5338.844 | 10706 | 34.39 | 0 |
| Height | 68.86 | + |  | + |  | + |  | + | 12 | -5341.045 | 10706.3 | 34.71 | 0 |
| Height | 89.01 | + | + |  | + |  | + | + | 13 | -5340.392 | 10707.1 | 35.44 | 0 |
| Height | 89.06 | + | + | + |  |  | + | + | 13 | -5340.401 | 10707.1 | 35.46 | 0 |
| Height | 68.97 | + |  |  | + | + |  | + | 12 | -5341.553 | 10707.4 | 35.72 | 0 |
| Height | 89.09 | + | + |  |  |  | + | + | 12 | -5341.786 | 10707.8 | 36.19 | 0 |
| Height | 69.02 | + |  |  |  | + |  | + | 11 | -5342.911 | 10708 | 36.4 | 0 |
| Height | 88.72 | + |  | + | + |  | + | + | 13 | -5340.909 | 10708.1 | 36.48 | 0 |
| Height | 88.82 | + |  | + |  |  | + | + | 12 | -5342.567 | 10709.4 | 37.75 | 0 |
| Height | 88.77 | + |  |  | + |  | + | + | 12 | -5342.637 | 10709.5 | 37.89 | 0 |
| Height | 88.85 | + |  |  |  |  | + | + | 11 | -5344.08 | 10710.4 | 38.74 | 0 |
| Height | 79.6 | + | + | + | + |  |  | + | 12 | -5356.043 | 10736.3 | 64.7 | 0 |
| Height | 79.72 | + | + | + |  |  |  | + | 11 | -5357.539 | 10737.3 | 65.65 | 0 |
| Height | 79.65 | + | + |  | + |  |  | + | 11 | -5357.641 | 10737.5 | 65.86 | 0 |
| Height | 79.73 | + | + |  |  |  |  | + | 10 | -5358.973 | 10738.1 | 66.49 | 0 |
| Height | 79.34 | + |  | + | + |  |  | + | 11 | -5358.175 | 10738.6 | 66.93 | 0 |
| Height | 79.46 | + |  | + |  |  |  | + | 10 | -5359.769 | 10739.7 | 68.08 | 0 |
| Height | 79.38 | + |  |  | + |  |  | + | 10 | -5359.977 | 10740.1 | 68.49 | 0 |
| Height | 79.47 | + |  |  |  |  |  | + | 9 | -5361.355 | 10740.9 | 69.22 | 0 |
| Height | 77.61 | + | + | + | + | + | + |  | 15 | -5377.554 | 10785.5 | 113.86 | 0 |
| Height | 77.68 | + | + | + |  | + | + |  | 14 | -5378.948 | 10786.2 | 114.6 | 0 |
| Height | 77.25 | + | + |  | + | + | + |  | 14 | -5380.098 | 10788.5 | 116.9 | 0 |
| Height | 77.3 | + | + |  |  | + | + |  | 13 | -5381.809 | 10789.9 | 118.28 | 0 |
| Height | 77.43 | + |  | + | + | + | + |  | 14 | -5380.907 | 10790.2 | 118.52 | 0 |
| Height | 77.51 | + |  | + |  | + | + |  | 13 | -5382.412 | 10791.1 | 119.48 | 0 |
| Height | 77.15 | + |  |  | + | + | + |  | 13 | -5383.017 | 10792.3 | 120.69 | 0 |
| Height | 77.21 | + |  |  |  | + | + |  | 12 | -5384.808 | 10793.9 | 122.23 | 0 |
| Height | 68.5 | + | + | + | + | + |  |  | 13 | -5394.676 | 10815.7 | 144.01 | 0 |
| Height | 68.57 | + | + | + |  | + |  |  | 12 | -5396.033 | 10816.3 | 144.68 | 0 |
| Height | 68.19 | + | + |  | + | + |  |  | 12 | -5397.133 | 10818.5 | 146.88 | 0 |
| Height | 68.26 | + | + |  |  | + |  |  | 11 | -5398.766 | 10819.7 | 148.11 | 0 |
| Height | 68.29 | + |  | + | + | + |  |  | 12 | -5398.127 | 10820.5 | 148.87 | 0 |
| Height | 68.38 | + |  | + |  | + |  |  | 11 | -5399.588 | 10821.4 | 149.75 | 0 |
| Height | 68.06 | + |  |  | + | + |  |  | 11 | -5400.156 | 10822.5 | 150.89 | 0 |
| Height | 68.13 | + |  |  |  | + |  |  | 10 | -5401.866 | 10823.9 | 152.27 | 0 |
| Height | 89.33 | + | + | + | + |  | + |  | 13 | -5399.175 | 10824.6 | 153.01 | 0 |
| Height | 89.41 | + | + | + |  |  | + |  | 12 | -5400.56 | 10825.4 | 153.74 | 0 |
| Height | 89.02 | + |  | + | + |  | + |  | 12 | -5401.826 | 10827.9 | 156.27 | 0 |
| Height | 89.11 | + |  | + |  |  | + |  | 11 | -5403.3 | 10828.8 | 157.18 | 0 |
| Height | 89.18 | + | + |  | + |  | + |  | 12 | -5402.336 | 10828.9 | 157.29 | 0 |
| Height | 89.3 | + | + |  |  |  | + |  | 11 | -5404.125 | 10830.5 | 158.83 | 0 |
| Height | 88.93 | + |  |  | + |  | + |  | 11 | -5404.541 | 10831.3 | 159.66 | 0 |
| Height | 89.06 | + |  |  |  |  | + |  | 10 | -5406.397 | 10833 | 161.33 | 0 |
| Height | 85.09 |  | + | + | + | + | + | + | 11 | -5406.245 | 10834.7 | 163.07 | 0 |
| Height | 85.49 |  |  | + | + | + | + | + | 10 | -5409.063 | 10838.3 | 166.67 | 0 |
| Height | 86.52 |  | + |  | + | + | + | + | 10 | -5414.004 | 10848.2 | 176.55 | 0 |
| Height | 86.83 |  |  |  | + | + | + | + | 9 | -5416.381 | 10850.9 | 179.27 | 0 |
| Height | 80 | + | + | + | + |  |  |  | 11 | -5416.22 | 10854.7 | 183.02 | 0 |
| Height | 80.08 | + | + | + |  |  |  |  | 10 | -5417.565 | 10855.3 | 183.67 | 0 |
| Height | 79.66 | + |  | + | + |  |  |  | 10 | -5418.959 | 10858.1 | 186.46 | 0 |
| Height | 79.89 | + | + |  | + |  |  |  | 10 | -5419.233 | 10858.6 | 187.01 | 0 |
| Height | 79.75 | + |  | + |  |  |  |  | 9 | -5420.387 | 10858.9 | 187.28 | 0 |
| Height | 80.03 | + | + |  |  |  |  |  | 9 | -5420.928 | 10860 | 188.36 | 0 |
| Height | 79.61 | + |  |  | + |  |  |  | 9 | -5421.53 | 10861.2 | 189.57 | 0 |
| Height | 86.99 |  | + | + |  | + | + | + | 10 | -5421.23 | 10862.6 | 191 | 0 |
| Height | 79.75 | + |  |  |  |  |  |  | 8 | -5423.29 | 10862.7 | 191.06 | 0 |
| Height | 95.2 |  | + | + | + |  | + | + | 9 | -5424.229 | 10866.6 | 194.97 | 0 |
| Height | 75.83 |  | + | + | + | + |  | + | 9 | -5424.389 | 10866.9 | 195.29 | 0 |
| Height | 76.25 |  |  | + | + | + |  | + | 8 | -5427.217 | 10870.6 | 198.91 | 0 |
| Height | 87.93 |  | + |  |  | + | + | + | 9 | -5426.209 | 10870.6 | 198.92 | 0 |
| Height | 87.9 |  |  | + |  | + | + | + | 9 | -5426.71 | 10871.6 | 199.93 | 0 |
| Height | 95.83 |  |  | + | + |  | + | + | 8 | -5427.734 | 10871.6 | 199.94 | 0 |
| Height | 88.66 |  |  |  |  | + | + | + | 8 | -5430.878 | 10877.9 | 206.23 | 0 |
| Diameter | 2.112 | + |  |  |  | + | + |  | 12 | 92.685 | -161.1 | 0 | 0.92 |
| Diameter | 2.112 | + |  |  | + | + | + |  | 13 | 90.18 | -154.1 | 7.05 | 0.027 |
| Diameter | 2.113 | + |  |  |  | + | + | + | 13 | 89.753 | -153.2 | 7.91 | 0.018 |
| Diameter | 2.113 | + | + |  |  | + | + |  | 13 | 89.605 | -152.9 | 8.2 | 0.015 |
| Diameter | 2.112 | + |  | + |  | + | + |  | 13 | 89.446 | -152.6 | 8.52 | 0.013 |
| Diameter | 2.006 | + |  |  |  |  | + |  | 10 | 85.266 | -150.4 | 10.76 | 0.004 |
| Diameter | 2.112 | + |  |  | + | + | + | + | 14 | 87.209 | -146.1 | 15.04 | 0 |
| Diameter | 2.112 | + | + |  | + | + | + |  | 14 | 87.07 | -145.8 | 15.32 | 0 |
| Diameter | 2.111 | + |  | + | + | + | + |  | 14 | 87.042 | -145.7 | 15.38 | 0 |
| Diameter | 2.113 | + | + |  |  | + | + | + | 14 | 86.669 | -145 | 16.12 | 0 |
| Diameter | 2.113 | + |  | + |  | + | + | + | 14 | 86.588 | -144.8 | 16.28 | 0 |
| Diameter | 2.113 | + | + | + |  | + | + |  | 14 | 86.372 | -144.4 | 16.72 | 0 |
| Diameter | 2.005 | + |  |  | + |  | + |  | 11 | 82.693 | -143.2 | 17.94 | 0 |
| Diameter | 2.008 | + | + |  |  |  | + |  | 11 | 82.448 | -142.7 | 18.43 | 0 |
| Diameter | 2.006 | + |  |  |  |  | + | + | 11 | 82.169 | -142.1 | 18.99 | 0 |
| Diameter | 2.006 | + |  | + |  |  | + |  | 11 | 82.105 | -142 | 19.12 | 0 |
| Diameter | 2.111 | + |  | + | + | + | + | + | 15 | 84.21 | -138 | 23.09 | 0 |
| Diameter | 2.113 | + | + |  | + | + | + | + | 15 | 84.096 | -137.8 | 23.32 | 0 |
| Diameter | 2.111 | + | + | + | + | + | + |  | 15 | 83.915 | -137.4 | 23.68 | 0 |
| Diameter | 2.113 | + | + | + |  | + | + | + | 15 | 83.491 | -136.6 | 24.53 | 0 |
| Diameter | 2.007 | + | + |  | + |  | + |  | 12 | 79.83 | -135.4 | 25.71 | 0 |
| Diameter | 2.005 | + |  | + | + |  | + |  | 12 | 79.733 | -135.2 | 25.9 | 0 |
| Diameter | 2.005 | + |  |  | + |  | + | + | 12 | 79.567 | -134.9 | 26.24 | 0 |
| Diameter | 2.008 | + | + |  |  |  | + | + | 12 | 79.347 | -134.4 | 26.68 | 0 |
| Diameter | 2.008 | + | + | + |  |  | + |  | 12 | 79.252 | -134.2 | 26.87 | 0 |
| Diameter | 2.006 | + |  | + |  |  | + | + | 12 | 79.13 | -134 | 27.11 | 0 |
| Diameter | 2.111 | + | + | + | + | + | + | + | 16 | 81.064 | -129.7 | 31.43 | 0 |
| Diameter | 2.006 | + | + | + | + |  | + |  | 13 | 76.796 | -127.3 | 33.82 | 0 |
| Diameter | 2.004 | + |  | + | + |  | + | + | 13 | 76.781 | -127.3 | 33.85 | 0 |
| Diameter | 2.007 | + | + |  | + |  | + | + | 13 | 76.701 | -127.1 | 34.01 | 0 |
| Diameter | 2.008 | + | + | + |  |  | + | + | 13 | 76.249 | -126.2 | 34.92 | 0 |
| Diameter | 2.006 | + | + | + | + |  | + | + | 14 | 73.817 | -119.3 | 41.82 | 0 |
| Diameter | 1.933 | + |  |  |  | + |  |  | 10 | 54.018 | -87.9 | 73.26 | 0 |
| Diameter | 1.829 | + |  |  |  |  |  |  | 8 | 51.095 | -86.1 | 75.04 | 0 |
| Diameter | 1.932 | + |  |  | + | + |  |  | 11 | 51.334 | -80.5 | 80.66 | 0 |
| Diameter | 1.934 | + |  |  |  | + |  | + | 11 | 51.1 | -80 | 81.13 | 0 |
| Diameter | 1.933 | + | + |  |  | + |  |  | 11 | 51.019 | -79.8 | 81.29 | 0 |
| Diameter | 1.932 | + |  | + |  | + |  |  | 11 | 50.806 | -79.4 | 81.72 | 0 |
| Diameter | 1.827 | + |  |  | + |  |  |  | 9 | 48.35 | -78.6 | 82.56 | 0 |
| Diameter | 1.831 | + | + |  |  |  |  |  | 9 | 48.265 | -78.4 | 82.73 | 0 |
| Diameter | 1.828 | + |  |  |  |  |  | + | 9 | 48.051 | -78 | 83.16 | 0 |
| Diameter | 1.829 | + |  | + |  |  |  |  | 9 | 47.961 | -77.8 | 83.34 | 0 |
| Diameter | 1.933 | + |  |  | + | + |  | + | 12 | 48.381 | -72.5 | 88.61 | 0 |
| Diameter | 1.932 | + | + |  | + | + |  |  | 12 | 48.302 | -72.3 | 88.77 | 0 |
| Diameter | 1.931 | + |  | + | + | + |  |  | 12 | 48.255 | -72.3 | 88.86 | 0 |
| Diameter | 1.934 | + | + |  |  | + |  | + | 12 | 48.095 | -71.9 | 89.18 | 0 |
| Diameter | 1.933 | + |  | + |  | + |  | + | 12 | 47.997 | -71.7 | 89.38 | 0 |
| Diameter | 1.933 | + | + | + |  | + |  |  | 12 | 47.799 | -71.3 | 89.77 | 0 |
| Diameter | 1.829 | + | + |  | + |  |  |  | 10 | 45.48 | -70.8 | 90.34 | 0 |
| Diameter | 1.827 | + |  | + | + |  |  |  | 10 | 45.409 | -70.6 | 90.48 | 0 |
| Diameter | 1.827 | + |  |  | + |  |  | + | 10 | 45.278 | -70.4 | 90.74 | 0 |
| Diameter | 1.83 | + | + |  |  |  |  | + | 10 | 45.218 | -70.3 | 90.86 | 0 |
| Diameter | 1.83 | + | + | + |  |  |  |  | 10 | 45.091 | -70 | 91.11 | 0 |
| Diameter | 1.828 | + |  | + |  |  |  | + | 10 | 45.06 | -69.9 | 91.17 | 0 |
| Diameter | 1.931 | + |  | + | + | + |  | + | 13 | 45.472 | -64.6 | 96.47 | 0 |
| Diameter | 1.933 | + | + |  | + | + |  | + | 13 | 45.345 | -64.4 | 96.72 | 0 |
| Diameter | 1.931 | + | + | + | + | + |  |  | 13 | 45.19 | -64.1 | 97.03 | 0 |
| Diameter | 1.933 | + | + | + |  | + |  | + | 13 | 44.962 | -63.6 | 97.49 | 0 |
| Diameter | 1.827 | + |  | + | + |  |  | + | 11 | 42.532 | -62.8 | 98.27 | 0 |
| Diameter | 1.829 | + | + | + | + |  |  |  | 11 | 42.465 | -62.7 | 98.4 | 0 |
| Diameter | 1.829 | + | + |  | + |  |  | + | 11 | 42.405 | -62.6 | 98.52 | 0 |
| Diameter | 1.83 | + | + | + |  |  |  | + | 11 | 42.16 | -62.1 | 99.01 | 0 |
| Diameter | 1.932 | + | + | + | + | + |  | + | 14 | 42.381 | -56.4 | 104.7 | 0 |
| Diameter | 1.828 | + | + | + | + |  |  | + | 12 | 39.558 | -54.9 | 106.25 | 0 |
| Diameter | 2.287 |  | + | + | + | + | + | + | 11 | -348.379 | 719 | 880.09 | 0 |
| Diameter | 2.153 |  | + | + | + |  | + | + | 9 | -358.579 | 735.3 | 896.42 | 0 |
| Diameter | 2.308 |  | + | + | + | + | + |  | 10 | -358.477 | 737.1 | 898.25 | 0 |
| Diameter | 2.325 |  | + |  | + | + | + | + | 10 | -361.214 | 742.6 | 903.72 | 0 |
| Diameter | 2.167 |  | + | + | + |  | + |  | 8 | -370.018 | 756.2 | 917.27 | 0 |
| Diameter | 2.179 |  | + |  | + |  | + | + | 8 | -374.556 | 765.2 | 926.34 | 0 |
| Diameter | 2.32 |  |  | + | + | + | + | + | 10 | -379.975 | 780.1 | 941.24 | 0 |
| Diameter | 2.373 |  | + |  | + | + | + |  | 9 | -383.425 | 785 | 946.11 | 0 |
| Diameter | 2.106 |  | + | + | + | + |  | + | 9 | -386.647 | 791.4 | 952.55 | 0 |
| Diameter | 2.194 |  |  | + | + |  | + | + | 8 | -388.131 | 792.4 | 953.49 | 0 |
| Diameter | 2.353 |  |  |  | + | + | + | + | 9 | -388.556 | 795.3 | 956.37 | 0 |
| Diameter | 1.972 |  | + | + | + |  |  | + | 7 | -392.435 | 799 | 960.08 | 0 |
| Leaf length | 3.383 | + | + | + | + |  | + |  | 13 | -2181.152 | 4388.6 | 0 | 0.821 |
| Leaf length | 3.381 | + | + | + | + |  | + | + | 14 | -2182.045 | 4392.4 | 3.83 | 0.121 |
| Leaf length | 3.469 | + | + | + | + | + | + |  | 15 | -2182.433 | 4395.3 | 6.66 | 0.029 |
| Leaf length | 3.438 | + |  | + | + |  | + |  | 12 | -2186.368 | 4397 | 8.39 | 0.012 |
| Leaf length | 3.137 | + | + | + | + |  |  |  | 11 | -2187.824 | 4397.9 | 9.26 | 0.008 |
| Leaf length | 3.473 | + | + | + | + | + | + | + | 16 | -2183.213 | 4398.9 | 10.27 | 0.005 |
| Leaf length | 3.437 | + |  | + | + |  | + | + | 13 | -2187.338 | 4401 | 12.37 | 0.002 |
| Leaf length | 3.135 | + | + | + | + |  |  | + | 12 | -2188.724 | 4401.7 | 13.1 | 0.001 |
| Leaf length | 3.509 | + |  | + | + | + | + |  | 14 | -2188.044 | 4404.4 | 15.83 | 0 |
| Leaf length | 3.218 | + | + | + | + | + |  |  | 13 | -2189.408 | 4405.1 | 16.51 | 0 |
| Leaf length | 3.439 | + | + | + |  |  | + |  | 12 | -2190.943 | 4406.1 | 17.54 | 0 |
| Leaf length | 3.191 | + |  | + | + |  |  |  | 10 | -2193.122 | 4406.4 | 17.82 | 0 |
| Leaf length | 3.513 | + |  | + | + | + | + | + | 15 | -2188.939 | 4408.3 | 19.67 | 0 |
| Leaf length | 3.222 | + | + | + | + | + |  | + | 14 | -2190.224 | 4408.8 | 20.19 | 0 |
| Leaf length | 3.438 | + | + | + |  |  | + | + | 13 | -2191.933 | 4410.2 | 21.56 | 0 |
| Leaf length | 3.19 | + |  | + | + |  |  | + | 11 | -2194.101 | 4410.4 | 21.82 | 0 |
| Leaf length | 3.486 | + |  | + |  |  | + |  | 11 | -2194.86 | 4411.9 | 23.33 | 0 |
| Leaf length | 3.526 | + | + | + |  | + | + |  | 14 | -2191.916 | 4412.2 | 23.58 | 0 |
| Leaf length | 3.256 | + |  | + | + | + |  |  | 12 | -2194.904 | 4414.1 | 25.46 | 0 |
| Leaf length | 3.194 | + | + | + |  |  |  |  | 10 | -2197.262 | 4414.7 | 26.1 | 0 |
| Leaf length | 3.53 | + | + | + |  | + | + | + | 15 | -2192.794 | 4416 | 27.38 | 0 |
| Leaf length | 3.485 | + |  | + |  |  | + | + | 12 | -2195.903 | 4416.1 | 27.46 | 0 |
| Leaf length | 3.26 | + |  | + | + | + |  | + | 13 | -2195.831 | 4418 | 29.36 | 0 |
| Leaf length | 3.193 | + | + | + |  |  |  | + | 11 | -2198.255 | 4418.7 | 30.12 | 0 |
| Leaf length | 3.56 | + |  | + |  | + | + |  | 13 | -2196.302 | 4418.9 | 30.3 | 0 |
| Leaf length | 3.24 | + |  | + |  |  |  |  | 9 | -2201.29 | 4420.7 | 32.12 | 0 |
| Leaf length | 3.275 | + | + | + |  | + |  |  | 12 | -2198.734 | 4421.7 | 33.12 | 0 |
| Leaf length | 3.564 | + |  | + |  | + | + | + | 14 | -2197.266 | 4422.9 | 34.27 | 0 |
| Leaf length | 3.239 | + |  | + |  |  |  | + | 10 | -2202.339 | 4424.9 | 36.25 | 0 |
| Leaf length | 3.279 | + | + | + |  | + |  | + | 13 | -2199.643 | 4425.6 | 36.98 | 0 |
| Leaf length | 3.307 | + |  | + |  | + |  |  | 11 | -2203.042 | 4428.3 | 39.7 | 0 |
| Leaf length | 3.311 | + |  | + |  | + |  | + | 12 | -2204.034 | 4432.3 | 43.72 | 0 |
| Leaf length | 3.091 |  | + | + | + |  | + |  | 8 | -2213.442 | 4443 | 54.4 | 0 |
| Leaf length | 3.1 |  | + | + | + |  | + | + | 9 | -2214.562 | 4447.3 | 58.67 | 0 |
| Leaf length | 3.201 |  | + | + | + | + | + |  | 10 | -2214.641 | 4449.5 | 60.86 | 0 |
| Leaf length | 2.858 |  | + | + | + |  |  |  | 6 | -2219.849 | 4451.8 | 63.16 | 0 |
| Leaf length | 3.217 |  | + | + | + | + | + | + | 11 | -2215.673 | 4453.6 | 64.96 | 0 |
| Leaf length | 2.867 |  | + | + | + |  |  | + | 7 | -2220.979 | 4456 | 67.45 | 0 |
| Leaf length | 2.963 |  | + | + | + | + |  |  | 8 | -2221.004 | 4458.1 | 69.52 | 0 |
| Leaf length | 3.199 |  |  | + | + |  | + |  | 7 | -2222.112 | 4458.3 | 69.71 | 0 |
| Leaf length | 2.979 |  | + | + | + | + |  | + | 9 | -2222.062 | 4462.3 | 73.67 | 0 |
| Leaf length | 3.206 |  |  | + | + |  | + | + | 8 | -2223.32 | 4462.8 | 74.15 | 0 |
| Leaf length | 3.296 |  |  | + | + | + | + |  | 9 | -2223.425 | 4465 | 76.39 | 0 |
| Leaf length | 2.964 |  |  | + | + |  |  |  | 5 | -2228.785 | 4467.6 | 79.02 | 0 |
| Leaf length | 3.308 |  |  | + | + | + | + | + | 10 | -2224.58 | 4469.3 | 80.74 | 0 |
| Leaf length | 2.971 |  |  | + | + |  |  | + | 6 | -2230.002 | 4472.1 | 83.47 | 0 |
| Leaf length | 3.055 |  |  | + | + | + |  |  | 7 | -2229.726 | 4473.5 | 84.94 | 0 |
| Leaf length | 3.067 |  |  | + | + | + |  | + | 8 | -2230.9 | 4477.9 | 89.31 | 0 |
| Leaf length | 3.298 | + | + |  | + |  | + | + | 13 | -2233.428 | 4493.2 | 104.55 | 0 |
| Leaf length | 3.309 |  | + | + |  |  | + |  | 7 | -2239.941 | 4494 | 105.37 | 0 |
| Leaf length | 3.315 |  | + | + |  |  | + | + | 8 | -2241.193 | 4498.5 | 109.9 | 0 |
| Leaf length | 3.08 | + | + |  | + |  |  | + | 11 | -2238.224 | 4498.7 | 110.06 | 0 |
| Leaf length | 3.444 |  | + | + |  | + | + |  | 9 | -2240.576 | 4499.3 | 110.7 | 0 |
| Leaf length | 3.265 | + | + |  | + | + | + | + | 15 | -2235.204 | 4500.8 | 112.2 | 0 |
| Leaf length | 3.299 | + | + |  | + |  | + |  | 12 | -2238.974 | 4502.2 | 113.6 | 0 |
| Leaf length | 3.074 |  | + | + |  |  |  |  | 5 | -2246.274 | 4502.6 | 113.99 | 0 |
| Leaf length | 3.455 |  | + | + |  | + | + | + | 10 | -2241.76 | 4503.7 | 115.1 | 0 |
| Leaf length | 3.047 | + | + |  | + | + |  | + | 13 | -2240.195 | 4506.7 | 118.09 | 0 |
| Leaf length | 3.079 |  | + | + |  |  |  | + | 6 | -2247.528 | 4507.1 | 118.52 | 0 |
| Leaf length | 3.081 | + | + |  | + |  |  |  | 10 | -2243.535 | 4507.2 | 118.65 | 0 |
| Leaf length | 3.204 |  | + | + |  | + |  |  | 7 | -2247.032 | 4508.2 | 119.55 | 0 |
| Leaf length | 3.231 | + | + |  | + | + | + |  | 14 | -2240.534 | 4509.4 | 120.81 | 0 |
| Leaf length | 3.214 |  | + | + |  | + |  | + | 8 | -2248.231 | 4512.6 | 123.98 | 0 |
| Leaf length | 3.014 | + | + |  | + | + |  |  | 12 | -2245.233 | 4514.7 | 126.12 | 0 |
| Leaf length | 3.374 | + |  |  | + |  | + | + | 12 | -2245.239 | 4514.7 | 126.13 | 0 |
| Leaf length | 3.156 | + |  |  | + |  |  | + | 10 | -2249.987 | 4520.2 | 131.55 | 0 |
| Leaf length | 3.479 |  |  | + |  |  | + |  | 6 | -2254.46 | 4521 | 132.38 | 0 |
| Leaf length | 3.306 | + |  |  | + | + | + | + | 14 | -2246.944 | 4522.2 | 133.63 | 0 |
| Leaf length | 3.377 | + |  |  | + |  | + |  | 11 | -2251.02 | 4524.3 | 135.65 | 0 |
| Leaf length | 3.481 |  |  | + |  |  | + | + | 7 | -2255.752 | 4525.6 | 136.99 | 0 |
| Leaf length | 3.601 |  |  | + |  | + | + |  | 8 | -2255.346 | 4526.8 | 138.21 | 0 |
| Leaf length | 3.088 | + |  |  | + | + |  | + | 12 | -2251.652 | 4527.6 | 138.96 | 0 |
| Leaf length | 3.159 | + |  |  | + |  |  |  | 9 | -2255.511 | 4529.2 | 140.57 | 0 |
| Leaf length | 3.242 |  |  | + |  |  |  |  | 4 | -2261.269 | 4530.6 | 141.97 | 0 |
| Leaf length | 3.271 | + |  |  | + | + | + |  | 13 | -2252.252 | 4530.8 | 142.2 | 0 |
| Leaf length | 3.607 |  |  | + |  | + | + | + | 9 | -2256.613 | 4531.4 | 142.77 | 0 |
| Leaf number | 69.02 | + | + | + | + | + | + | + | 16 | -5635.848 | 11304.1 | 0 | 0.672 |
| Leaf number | 69.27 | + |  | + | + | + | + | + | 15 | -5638.478 | 11307.4 | 3.21 | 0.135 |
| Leaf number | 68 | + | + | + | + |  | + | + | 14 | -5639.71 | 11307.8 | 3.62 | 0.11 |
| Leaf number | 69.46 | + | + | + |  | + | + | + | 15 | -5639.683 | 11309.8 | 5.62 | 0.041 |
| Leaf number | 68.38 | + |  | + | + |  | + | + | 13 | -5642.28 | 11310.9 | 6.72 | 0.023 |
| Leaf number | 69.68 | + |  | + |  | + | + | + | 14 | -5642.106 | 11312.6 | 8.41 | 0.01 |
| Leaf number | 68.44 | + | + | + |  |  | + | + | 13 | -5643.553 | 11313.4 | 9.26 | 0.007 |
| Leaf number | 68.77 | + |  | + |  |  | + | + | 12 | -5645.903 | 11316.1 | 11.92 | 0.002 |
| Leaf number | 68.75 | + | + | + | + | + | + |  | 15 | -5644.521 | 11319.4 | 15.29 | 0 |
| Leaf number | 68.99 | + |  | + | + | + | + |  | 14 | -5646.954 | 11322.3 | 18.11 | 0 |
| Leaf number | 68.15 | + | + | + | + |  | + |  | 13 | -5648.225 | 11322.8 | 18.61 | 0 |
| Leaf number | 69.18 | + | + | + |  | + | + |  | 14 | -5648.151 | 11324.6 | 20.5 | 0 |
| Leaf number | 68.51 | + |  | + | + |  | + |  | 12 | -5650.64 | 11325.5 | 21.39 | 0 |
| Leaf number | 69.38 | + |  | + |  | + | + |  | 13 | -5650.407 | 11327.1 | 22.97 | 0 |
| Leaf number | 68.58 | + | + | + |  |  | + |  | 12 | -5651.849 | 11328 | 23.81 | 0 |
| Leaf number | 68.86 |  | + | + | + | + | + | + | 11 | -5653.114 | 11328.4 | 24.3 | 0 |
| Leaf number | 68.87 | + |  | + |  |  | + |  | 11 | -5654.072 | 11330.4 | 26.22 | 0 |
| Leaf number | 64.81 | + | + | + | + | + |  | + | 14 | -5651.063 | 11330.5 | 26.33 | 0 |
| Leaf number | 69.4 |  |  | + | + | + | + | + | 10 | -5655.875 | 11331.9 | 27.79 | 0 |
| Leaf number | 67.74 |  | + | + | + |  | + | + | 9 | -5657.087 | 11332.3 | 28.18 | 0 |
| Leaf number | 65.07 | + |  | + | + | + |  | + | 13 | -5653.677 | 11333.7 | 29.51 | 0 |
| Leaf number | 63.81 | + | + | + | + |  |  | + | 12 | -5655.287 | 11334.8 | 30.69 | 0 |
| Leaf number | 65.25 | + | + | + |  | + |  | + | 13 | -5654.779 | 11335.9 | 31.71 | 0 |
| Leaf number | 68.39 |  |  | + | + |  | + | + | 8 | -5659.876 | 11335.9 | 31.73 | 0 |
| Leaf number | 64.2 | + |  | + | + |  |  | + | 11 | -5657.941 | 11338.1 | 33.95 | 0 |
| Leaf number | 65.46 | + |  | + |  | + |  | + | 12 | -5657.194 | 11338.6 | 34.5 | 0 |
| Leaf number | 67.79 |  | + | + | + | + | + |  | 10 | -5659.699 | 11339.6 | 35.43 | 0 |
| Leaf number | 64.24 | + | + | + |  |  |  | + | 11 | -5658.902 | 11340 | 35.88 | 0 |
| Leaf number | 70.65 |  | + | + |  | + | + | + | 10 | -5660.237 | 11340.7 | 36.51 | 0 |
| Leaf number | 68.3 |  |  | + | + | + | + |  | 9 | -5662.234 | 11342.6 | 38.47 | 0 |
| Leaf number | 64.57 | + |  | + |  |  |  | + | 10 | -5661.341 | 11342.9 | 38.72 | 0 |
| Leaf number | 67.03 |  | + | + | + |  | + |  | 8 | -5663.527 | 11343.2 | 39.03 | 0 |
| Leaf number | 69.34 |  | + | + |  |  | + | + | 8 | -5664.302 | 11344.7 | 40.58 | 0 |
| Leaf number | 64.53 | + | + | + | + | + |  |  | 13 | -5659.562 | 11345.4 | 41.28 | 0 |
| Leaf number | 67.64 |  |  | + | + |  | + |  | 7 | -5666.113 | 11346.3 | 42.17 | 0 |
| Leaf number | 71.61 |  |  | + |  | + | + | + | 9 | -5664.19 | 11346.5 | 42.38 | 0 |
| Leaf number | 64.77 | + |  | + | + | + |  |  | 12 | -5661.981 | 11348.2 | 44.08 | 0 |
| Leaf number | 63.94 | + | + | + | + |  |  |  | 11 | -5663.713 | 11349.6 | 45.5 | 0 |
| Leaf number | 64.96 | + | + | + |  | + |  |  | 12 | -5663.08 | 11350.4 | 46.27 | 0 |
| Leaf number | 70.43 |  |  | + |  |  | + | + | 7 | -5668.277 | 11350.6 | 46.5 | 0 |
| Leaf number | 69.55 |  | + | + |  | + | + |  | 9 | -5666.401 | 11351 | 46.81 | 0 |
| Leaf number | 64.3 | + |  | + | + |  |  |  | 10 | -5666.203 | 11352.6 | 48.44 | 0 |
| Leaf number | 65.15 | + |  | + |  | + |  |  | 11 | -5665.331 | 11352.9 | 48.73 | 0 |
| Leaf number | 64.35 | + | + | + |  |  |  |  | 10 | -5667.124 | 11354.4 | 50.28 | 0 |
| Leaf number | 64.82 |  | + | + | + | + |  | + | 9 | -5668.203 | 11354.6 | 50.41 | 0 |
| Leaf number | 68.61 |  | + | + |  |  | + |  | 7 | -5670.288 | 11354.7 | 50.52 | 0 |
| Leaf number | 70.49 |  |  | + |  | + | + |  | 8 | -5670.002 | 11356.1 | 51.98 | 0 |
| Leaf number | 64.66 | + |  | + |  |  |  |  | 9 | -5669.427 | 11357 | 52.86 | 0 |
| Leaf number | 65.35 |  |  | + | + | + |  | + | 8 | -5670.984 | 11358.1 | 53.94 | 0 |
| Leaf number | 63.7 |  | + | + | + |  |  | + | 7 | -5672.775 | 11359.6 | 55.5 | 0 |
| Leaf number | 69.65 |  |  | + |  |  | + |  | 6 | -5673.942 | 11360 | 55.81 | 0 |
| Leaf number | 64.38 |  |  | + | + |  |  | + | 6 | -5675.747 | 11363.6 | 59.42 | 0 |
| Leaf number | 63.75 |  | + | + | + | + |  |  | 8 | -5674.609 | 11365.3 | 61.19 | 0 |
| Leaf number | 66.58 |  | + | + |  | + |  | + | 8 | -5675.328 | 11366.8 | 62.63 | 0 |
| Leaf number | 64.26 |  |  | + | + | + |  |  | 7 | -5677.164 | 11368.4 | 64.28 | 0 |
| Leaf number | 62.99 |  | + | + | + |  |  |  | 6 | -5679.081 | 11370.2 | 66.09 | 0 |
| Leaf number | 65.27 |  | + | + |  |  |  | + | 6 | -5679.907 | 11371.9 | 67.74 | 0 |
| Leaf number | 67.54 |  |  | + |  | + |  | + | 7 | -5679.329 | 11372.8 | 68.61 | 0 |
| Leaf number | 63.63 |  |  | + | + |  |  |  | 5 | -5681.834 | 11373.7 | 69.57 | 0 |
| Leaf number | 65.49 |  | + | + |  | + |  |  | 7 | -5681.32 | 11376.7 | 72.59 | 0 |
| Leaf number | 66.4 |  |  | + |  |  |  | + | 5 | -5684.146 | 11378.3 | 74.2 | 0 |
| Leaf number | 64.53 |  | + | + |  |  |  |  | 5 | -5685.769 | 11381.6 | 77.44 | 0 |
| Leaf number | 66.43 |  |  | + |  | + |  |  | 6 | -5684.968 | 11382 | 77.86 | 0 |
| Leaf number | 65.62 |  |  | + |  |  |  |  | 4 | -5689.668 | 11387.4 | 83.23 | 0 |
| Leaf number | 63.27 | + | + |  | + | + | + | + | 15 | -5761.973 | 11554.3 | 250.2 | 0 |
| Leaf number | 65.71 | + | + |  | + |  | + | + | 13 | -5767.119 | 11560.5 | 256.39 | 0 |
| Leaf number | 63.77 | + |  |  | + | + | + | + | 14 | -5769.466 | 11567.3 | 263.13 | 0 |
| Leaf number | 60.03 | + | + |  | + | + |  | + | 13 | -5773.973 | 11574.2 | 270.1 | 0 |
| Leaf number | 66.7 | + |  |  | + |  | + | + | 12 | -5775.307 | 11574.9 | 270.73 | 0 |
| Leaf number | 62.38 | + | + |  | + |  |  | + | 11 | -5779.424 | 11581.1 | 276.92 | 0 |
| Leaf number | 64.11 | + | + |  |  | + | + | + | 14 | -5777.041 | 11582.4 | 278.28 | 0 |
| Leaf number | 60.54 | + |  |  | + | + |  | + | 12 | -5781.316 | 11586.9 | 282.75 | 0 |
| Leaf number | 66.79 | + | + |  |  |  | + | + | 12 | -5782.477 | 11589.2 | 285.07 | 0 |
| Leaf number | 59.13 |  | + |  | + | + | + | + | 10 | -5786.067 | 11592.3 | 288.17 | 0 |
| Leaf number | 64.56 | + |  |  |  | + | + | + | 13 | -5783.663 | 11593.6 | 289.48 | 0 |
| Leaf number | 63.37 | + |  |  | + |  |  | + | 10 | -5787.648 | 11595.5 | 291.33 | 0 |
| Leaf width | 32.37 | + | + | + | + | + | + | + | 16 | -4910.178 | 9852.8 | 0 | 0.401 |
| Leaf width | 32.49 | + |  | + | + | + | + | + | 15 | -4911.904 | 9854.2 | 1.4 | 0.2 |
| Leaf width | 31.86 | + | + | + | + |  | + | + | 14 | -4912.99 | 9854.3 | 1.52 | 0.188 |
| Leaf width | 32.03 | + |  | + | + |  | + | + | 13 | -4914.61 | 9855.5 | 2.71 | 0.103 |
| Leaf width | 32.61 | + | + | + |  | + | + | + | 15 | -4913.355 | 9857.1 | 4.3 | 0.047 |
| Leaf width | 32.71 | + |  | + |  | + | + | + | 14 | -4914.906 | 9858.2 | 5.35 | 0.028 |
| Leaf width | 32.1 | + | + | + |  |  | + | + | 13 | -4916.243 | 9858.8 | 5.98 | 0.02 |
| Leaf width | 32.24 | + |  | + |  |  | + | + | 12 | -4917.685 | 9859.6 | 6.82 | 0.013 |
| Leaf width | 30.21 | + | + | + | + | + |  | + | 14 | -4922.178 | 9872.7 | 19.9 | 0 |
| Leaf width | 29.71 | + | + | + | + |  |  | + | 12 | -4924.881 | 9874 | 21.21 | 0 |
| Leaf width | 30.32 | + |  | + | + | + |  | + | 13 | -4923.878 | 9874.1 | 21.25 | 0 |
| Leaf width | 29.89 | + |  | + | + |  |  | + | 11 | -4926.548 | 9875.3 | 22.51 | 0 |
| Leaf width | 30.44 | + | + | + |  | + |  | + | 13 | -4925.259 | 9876.8 | 24.01 | 0 |
| Leaf width | 32.18 | + | + | + | + | + | + |  | 15 | -4923.536 | 9877.5 | 24.66 | 0 |
| Leaf width | 30.54 | + |  | + |  | + |  | + | 12 | -4926.793 | 9877.8 | 25.04 | 0 |
| Leaf width | 29.95 | + | + | + |  |  |  | + | 11 | -4927.946 | 9878.1 | 25.3 | 0 |
| Leaf width | 32.29 | + |  | + | + | + | + |  | 14 | -4925.044 | 9878.4 | 25.63 | 0 |
| Leaf width | 31.97 | + | + | + | + |  | + |  | 13 | -4926.121 | 9878.5 | 25.74 | 0 |
| Leaf width | 30.1 | + |  | + |  |  |  | + | 10 | -4929.438 | 9879.1 | 26.25 | 0 |
| Leaf width | 32.12 | + |  | + | + |  | + |  | 12 | -4927.574 | 9879.4 | 26.6 | 0 |
| Leaf width | 32.41 | + | + | + |  | + | + |  | 14 | -4926.434 | 9881.2 | 28.41 | 0 |
| Leaf width | 32.49 | + |  | + |  | + | + |  | 13 | -4927.806 | 9881.9 | 29.11 | 0 |
| Leaf width | 32.2 | + | + | + |  |  | + |  | 12 | -4929.075 | 9882.4 | 29.6 | 0 |
| Leaf width | 32.32 | + |  | + |  |  | + |  | 11 | -4930.386 | 9883 | 30.18 | 0 |
| Leaf width | 30.01 | + | + | + | + | + |  |  | 13 | -4935.296 | 9896.9 | 44.09 | 0 |
| Leaf width | 30.11 | + |  | + | + | + |  |  | 12 | -4936.784 | 9897.8 | 45.02 | 0 |
| Leaf width | 29.81 | + | + | + | + |  |  |  | 11 | -4937.875 | 9898 | 45.16 | 0 |
| Leaf width | 29.96 | + |  | + | + |  |  |  | 10 | -4939.369 | 9898.9 | 46.11 | 0 |
| Leaf width | 30.23 | + | + | + |  | + |  |  | 12 | -4938.109 | 9900.5 | 47.67 | 0 |
| Leaf width | 30.31 | + |  | + |  | + |  |  | 11 | -4939.467 | 9901.2 | 48.35 | 0 |
| Leaf width | 30.03 | + | + | + |  |  |  |  | 10 | -4940.659 | 9901.5 | 48.69 | 0 |
| Leaf width | 30.16 | + |  | + |  |  |  |  | 9 | -4942.013 | 9902.2 | 49.37 | 0 |
| Leaf width | 35.73 |  | + | + | + | + | + | + | 11 | -4956.071 | 9934.4 | 81.55 | 0 |
| Leaf width | 35.09 |  | + | + | + |  | + | + | 9 | -4958.885 | 9935.9 | 83.11 | 0 |
| Leaf width | 35.28 |  | + | + | + | + | + |  | 10 | -4959.878 | 9939.9 | 87.13 | 0 |
| Leaf width | 36.22 |  |  | + | + | + | + | + | 10 | -4960.351 | 9940.9 | 88.08 | 0 |
| Leaf width | 34.8 |  | + | + | + |  | + |  | 8 | -4962.565 | 9941.2 | 88.44 | 0 |
| Leaf width | 35.67 |  |  | + | + |  | + | + | 8 | -4963.079 | 9942.3 | 89.47 | 0 |
| Leaf width | 35.76 |  |  | + | + | + | + |  | 9 | -4963.882 | 9945.9 | 93.11 | 0 |
| Leaf width | 35.36 |  |  | + | + |  | + |  | 7 | -4966.518 | 9947.1 | 94.32 | 0 |
| Leaf width | 36.87 |  | + | + |  | + | + | + | 10 | -4964.283 | 9948.7 | 95.94 | 0 |
| Leaf width | 36.12 |  | + | + |  |  | + | + | 8 | -4967.247 | 9950.6 | 97.81 | 0 |
| Leaf width | 36.41 |  | + | + |  | + | + |  | 9 | -4967.734 | 9953.6 | 100.81 | 0 |
| Leaf width | 33.62 |  | + | + | + | + |  | + | 9 | -4968.219 | 9954.6 | 101.78 | 0 |
| Leaf width | 35.81 |  | + | + |  |  | + |  | 7 | -4970.547 | 9955.2 | 102.38 | 0 |
| Leaf width | 32.98 |  | + | + | + |  |  | + | 7 | -4971.214 | 9956.5 | 103.71 | 0 |
| Leaf width | 37.68 |  |  | + |  | + | + | + | 9 | -4970.657 | 9959.5 | 106.66 | 0 |
| Leaf width | 33.16 |  | + | + | + | + |  |  | 8 | -4971.892 | 9959.9 | 107.1 | 0 |
| Leaf width | 37.02 |  |  | + |  |  | + | + | 7 | -4973.483 | 9961.1 | 108.25 | 0 |
| Leaf width | 34.1 |  |  | + | + | + |  | + | 8 | -4972.498 | 9961.1 | 108.31 | 0 |
| Leaf width | 32.68 |  | + | + | + |  |  |  | 6 | -4974.79 | 9961.6 | 108.84 | 0 |
| Leaf width | 33.57 |  |  | + | + |  |  | + | 6 | -4975.632 | 9963.3 | 110.53 | 0 |
| Leaf width | 37.23 |  |  | + |  | + | + |  | 8 | -4973.733 | 9963.6 | 110.78 | 0 |
| Leaf width | 36.71 |  |  | + |  |  | + |  | 6 | -4976.45 | 9965 | 112.16 | 0 |
| Leaf width | 33.65 |  |  | + | + | + |  |  | 7 | -4975.9 | 9965.9 | 113.09 | 0 |
| Leaf width | 33.26 |  |  | + | + |  |  |  | 5 | -4978.96 | 9968 | 115.16 | 0 |
| Leaf width | 34.74 |  | + | + |  | + |  | + | 8 | -4976.454 | 9969 | 116.22 | 0 |
| Leaf width | 33.99 |  | + | + |  |  |  | + | 6 | -4979.517 | 9971.1 | 118.3 | 0 |
| Leaf width | 34.29 |  | + | + |  | + |  |  | 7 | -4979.779 | 9973.6 | 120.84 | 0 |
| Leaf width | 33.68 |  | + | + |  |  |  |  | 5 | -4982.723 | 9975.5 | 122.69 | 0 |
| Leaf width | 35.54 |  |  | + |  | + |  | + | 7 | -4982.859 | 9979.8 | 127 | 0 |
| Leaf width | 34.91 |  |  | + |  |  |  | + | 5 | -4986.064 | 9982.2 | 129.37 | 0 |
| Leaf width | 35.1 |  |  | + |  | + |  |  | 6 | -4985.813 | 9983.7 | 130.89 | 0 |
| Leaf width | 34.6 |  |  | + |  |  |  |  | 4 | -4988.929 | 9985.9 | 133.09 | 0 |
| Leaf width | 29.33 | + | + |  | + | + | + | + | 15 | -5030.86 | 10092.1 | 239.31 | 0 |
| Leaf width | 30.65 | + | + |  | + |  | + | + | 13 | -5034.793 | 10095.9 | 243.08 | 0 |
| Leaf width | 29.58 | + |  |  | + | + | + | + | 14 | -5037.069 | 10102.5 | 249.68 | 0 |
| Leaf width | 27.65 | + | + |  | + | + |  | + | 13 | -5040.124 | 10106.5 | 253.74 | 0 |
| Leaf width | 31.16 | + |  |  | + |  | + | + | 12 | -5041.447 | 10107.1 | 254.34 | 0 |
| Leaf width | 28.94 | + | + |  | + |  |  | + | 11 | -5043.975 | 10110.2 | 257.36 | 0 |
| Leaf width | 27.91 | + |  |  | + | + |  | + | 12 | -5046.179 | 10116.6 | 263.81 | 0 |
| Leaf width | 29.76 | + | + |  |  | + | + | + | 14 | -5045.009 | 10118.4 | 265.56 | 0 |
| Leaf width | 29.45 | + |  |  | + |  |  | + | 10 | -5050.631 | 10121.4 | 268.64 | 0 |
| Leaf width | 31.23 | + | + |  |  |  | + | + | 12 | -5049.406 | 10123.1 | 270.26 | 0 |
| Leaf width | 29.99 | + |  |  |  | + | + | + | 13 | -5050.437 | 10127.2 | 274.37 | 0 |
| Leaf width | 28.12 | + | + |  |  | + |  | + | 12 | -5053.948 | 10132.2 | 279.35 | 0 |
